# Uncertainty reduction for precipitation prediction in North America

**DOI:** 10.1371/journal.pone.0301759

**Published:** 2024-05-22

**Authors:** Dan Lou, Wouter R. Berghuijs, Waheed Ullah, Boyuan Zhu, Dawei Shi, Yong Hu, Chao Li, Safi Ullah, Hao Zhou, Yuanfang Chai, Danyang Yu

**Affiliations:** 1 Nanjing Nriet Industrial Co., Ltd., Nanjing, China; 2 Department of Earth Sciences, Free University Amsterdam, Amsterdam, Netherlands; 3 Defense and Security, Rabdan Academy, Abu Dhabi, United Arab Emirates; 4 School of Hydraulic Engineering, Changsha University of Science & Technology, Changsha, China; 5 Key Laboratory of Water-Sediment Sciences and Water Disaster Prevention of Hunan Province, Changsha, China; 6 Lianyungang Meteorological Bureau, Haizhou District, Lianyungang City, Jiangsu Province, P.R. China; 7 State Key Laboratory of Water Resources and Hydropower Engineering Science, School of Water Resources and Hydropower Engineering, Wuhan University, Wuhan, China; 8 Jiangsu Meteorological Observatory, Nanjing, China; 9 Department of Atmosphere and Oceanic Sciences & Institute of Atmospheric Science, Fudan University, Shanghai, China; 10 Peixian Meteorological Bureau, Jiangsu Province, China; 11 Faculty of Geographical Science, State Key Laboratory of Earth Surface Processes and Resource Ecology, Beijing Normal University, Beijing, China; 12 Department of Water Resources, ITC Faculty of Geo-Information Science and Earth Observation, University of Twente, Enschede, The Netherlands; Instituto Nacional de Pesquisas Espaciais, BRAZIL

## Abstract

Large differences in projected future annual precipitation increases in North America exists across 27 CMIP6 models under four emission scenarios. These differences partly arise from weak representations of land-atmosphere interactions. Here we demonstrate an emergent constraint relationship between annual growth rates of future precipitation and growth rates of historical temperature. The original CMIP6 projections show 0.49% (SSP126), 0.98% (SSP245), 1.45% (SSP370) and 1.92% (SSP585) increases in precipitation per decade. Combining observed warming trends, the constrained results show that the best estimates of future precipitation increases are more likely to reach 0.40–0.48%, 0.83–0.93%, 1.29–1.45% and 1.70–1.87% respectively, implying an overestimated future precipitation increases across North America. The constrained results also are narrow the corresponding uncertainties (standard deviations) by 13.8–31.1%. The overestimated precipitation growth rates also reveal an overvalued annual growth rates in temperature (6.0–13.2% or 0.12–0.37°C) and in total evaporation (4.8–14.5%) by the original models’ predictions. These findings highlight the important role of temperature for accurate climate predictions, which is important as temperature from current climate models’ simulations often still have systematic errors.

## Introduction

The climate of North America varies due to changes in latitude, and a range of geographic features (including mountains and deserts), ranging from the frost-free tropical of southernmost Florida to the perennial ice and snow of the northernmost islands of the Greenland. Generally, on the mainland, the climate of becomes warmer the further south one travels, and drier the further west, until one reaches the West Coast. The annual mean precipitation, temperature and evapotranspiration are 562 mm, -3.2°C and 342 mm, respectively. The current population of Northern America is around 0.38 billion. However, North America has experienced severe droughts in recent decades [1,2], which has negatively influenced agriculture, energy production, food security, forestry, drinking water, and tourism [3–5]. Typically, the main drivers of drought are below-average precipitation and/or above-average temperature and evaporation [6]. Thereby, accurate predictions of future precipitation, temperature and total evaporation are crucial for mitigating the climate-driven drought risks [4].

Earth System models (ESMs) are widely used to investigate past climate variations and future climate predictions in response to various radiative forcings [7]. Despite consideration of physical, chemical, and biological processes, ESMs often poorly predict the most basic quantities, such precipitation, temperature, and evaporation, as seen in a series of publications of the Fifth Phase of the Coupled Model Intercomparison Project (CMIP5) [8–10]. For example, the spread of future increases in global mean surface temperatures during 2081–2100 across CMIP5 models is large, ranging from 0.3°C to 1.7°C (RCP2.6), 1.1°C to 2.6°C (RCP4.5), 1.4°C to 3.1°C (RCP6.0) and 2.6°C to 4.8°C (RCP8.5) [11]. The future global mean precipitation feedbacks with temperature also exhibits substantial uncertainties (0.5–4% °C^-1^) [11]. The new generation of ESMs (CMIP6) that has higher horizontal-vertical resolutions and more comprehensive experimental designs [12], still yields considerable uncertainties in predicting the basic climate variables [13–15]. This inaccurate information makes planning climate mitigation and adaptation measures more challenging [16].

In recent years, the emergent constraint technique that is based on the significant statistical relationship between simulated changes of historical climate variable *X* and predicted changes of future climate variable *Y* across an ESM ensemble has emerged [17–22] (See methods). This empirical relationship, combined with observed changes of climate variable *X*, has successfully reduced uncertainties in predicted changes of future climate variable *Y* (e.g., permafrost melt [23,24], marine primary production [25], Arctic sea-ice albedo feedback [26] and precipitation extremes [27]).

The key of the emergent constraint technique is to explore the mechanisms that underpin the emergent constraint relationship [16]. Thereby, we first explore the main driving factor (i.e. variable *x*) which dominates the large spread of predicted future annual precipitation growth rates (i.e. variable *y*) across the 27 CMIP6 models. The identified factor then is used to build the emergent constraint relationship with future annual precipitation growth rates across the CMIP6 models under scenarios SSP126, SSP245, SSP370 and SSP585. SSP126 represents the sustainable and “green” pathway with minimizing material resource and energy usage. SSP245 is the medium pathway that the world follows a path in which social, economic, and technological trends do not shift markedly from historical patterns. SSP370 has the high GHG emissions that a low international priority for addressing environmental concerns leads to strong environmental degradation in some regions. SSP585 has a very high GHG emissions (CO_2_ emissions triple by 2075) that inequality is rising. Some regions suffer drastic environmental damage. By combing the observations, we aim to reduce the uncertainty of future annual precipitation in North America during 2015–2100. To verify the robustness of the emergent constraint relationships from CMIP6, 33 CMIP5 models under RCP45 and RCP85 are also used for cross-checking of the constraint relationship on a new model ensemble, using exactly the same constraint processes. Finally, the constrained future precipitation changes, with smaller uncertainties relative to the raw model predictions, are used to re-estimate growth rates of future temperature and evapotranspiration in North America.

## Materials and methods

### Emergent constraint technique

In this study, we built the emergent constraint relationships between annual growth rates of simulated historical temperature and that of future precipitation across the CMIP6 and CMIP5 models (S1 and S2 Tables), by using the least-squares linear regression method (Eq 1). The prediction error of the regression (*σ*_*y*_) is estimated by Eq 2, following the method from Cox et al [21] and Chai et al [22]. Combining the observed temperature from four different data sets, the emergent relationship provides a tight constraint on future annual precipitation growth rates.

$$y_{i}=ax_{i}+b$$ (1)

where *y*_*i*_ (future predicted climate variable *y*, i.e., future annual precipitation growth rates) is the value given by *x*_*i*_ (historical simulated climate variable *x*, i.e., simulated historical annual temperature growth rates); *a* and *b* are the slope and intercept values, respectively;

$$\sigma_{y}\left(x\right)=s\sqrt{1+\frac{1}{N}+\frac{{\left(x-\bar{x}\right)}^{2}}{N\cdot \sigma_{x}^{2}}}$$ (2)

where *s* is used for minimizing the least-squares error, calculating by Eq 3; *N* is the number of models. *σ*_*x*_ is the variance of *x*_*i*_ Eq 4; $\bar{x}$ is the mean value;

$$s^{2}=\frac{1}{N-2}\sum_{n=1}^{N}{\left(y-y_{i}\right)}^{2}$$ (3)

$$\sigma_{x}=\sqrt{\sum_{n=1}^{N}{\left(x_{i}-\bar{x}\right)}^{2}/N}$$ (4)

### Calculation of probability density function (PDF)

For checking how significant the changes of future annual precipitation growth rates before and after applying the emergent constraint technique, we estimated the PDFs of the future annual precipitation growth rates before applying the technique (Eq 5). After the constraint, the PDFs for the constrained future annual precipitation growth rates (*PDF(F)*) is calculated by numerically integrating *PDF(F/ob)* and *PDF(ob)* (Eq 6).

$$PDF\left(y/x\right)=\frac{1}{\sqrt{2\pi \cdot \sigma_{y}^{2}}}\text{exp}\left\{-\frac{{\left(y-f\left(x\right)\right)}^{2}}{2\sigma_{y}^{2}}\right\}$$ (5)

where *PDF(y/x)* is the PDF around the best-fit linear regression, representing the PDF of *y* given *x*.

$$PDF\left(F\right)=\int_{-\infty }^{+\infty }PDF\left(F/ob\right)\cdot PDF\left(ob\right)\cdot dob$$ (6)

where *PDF(F/ob)* is the probability density for the “future climate projected variable” given the “historical observable variable”; and *PDF(ob)* is the observation-based PDF for “historical observable variable”.

### Clausius–Clapeyron relation

The Clausius–Clapeyron relations (Eqs 7 and 8) indicate that the saturation specific humidity (*q*_*s*_) increases by about 7% per degree of warming, i.e. α = 7% K^−1^ [28].

$$\frac{dq_{s}}{dT}=\frac{q_{s}\cdot L_{v}}{R_{v}\cdot T^{2}}$$ (7)

$$\frac{\Delta q_{s}}{q_{s}}=\frac{L_{v}}{R_{v}\cdot T^{2}}\Delta T\;=\alpha \cdot \Delta T$$ (8)

Where *q*_*s*_ and *T* are the saturation specific humidity and the temperature, respectively. *L*_*v*_ and *R*_*v*_ are the latent heat of condensation at temperature *T* and the gas constant for water vapour (461.5 J kg^−1^ K^−1^), respectively. Here we assumed that *Lv* = 2.5 10^6^ J kg^−1^ and the total pressure is much larger than the water vapour pressure.

### Thermodynamic equations indicating a linear relationship between precipitation and temperature

Held and Soden [29] proposed a thermodynamic scaling where precipitation (*Pre*) can be approximated as a product of convective mass flux (*M*_*f*_) and specific humidity (*q*), near the global land surface (Eq 9). Thus, combining Eqs 8 and 9, we can further obtain the Eq 10 [28]. Under a unchanged atmospheric circulation (Δ*M*_*f*_ = 0), Eq 10 shows a linear positive relationship between precipitation and temperature, i.e. per increase in temperature will lead to 7% increase in precipitation.


$$Pre=M_{f}\cdot q$$ (9)

$$\frac{\Delta Pre}{Pre}=\frac{\Delta M_{f}}{M_{f}}+\frac{\Delta q_{s}}{q_{s}}=\frac{\Delta M_{f}}{M_{f}}+0.07\cdot \Delta T$$ (10)

### Global land hydrological budget equation

The changes in precipitation (*ΔP*) are normally accompanied by coinciding changes in land surface runoff (*ΔR*), total evaporation (*ΔET*), soil water storage (*ΔSW*). These closely connected processes make up the land water cycle, which is described by Eq 11 [30–32].

ΔP=ΔR+ΔET+ΔSW+ε (11)

*Ɛ* is the other minor components in land water cycle (e.g., snow melting and human water uses).

### Multiple regression technique

To estimate the relative contribution of each potential driving factor (i.e., temperature, land surface runoff, soil water content and total evaporation) on the future precipitation changes, we used the multiple regression method that has been widely applied by previous studies [33–36]. After building the multiple regression relationships (Eq 12) between future precipitation changes and the physically relevant observed driving factors, we can obtain the regression coefficient of each driving factor (Eq 12). Then, the regression coefficients are used to estimate the standardized coefficient of each potential driving factor (Eq 13).

$$P=\beta_{1}\cdot R+\beta_{2}\cdot ET+\beta_{3}\cdot SW+\beta_{4}\cdot T+\varepsilon$$ (12)

$$Stc\_R=\beta_{1}\cdot \frac{std\left(R\right)}{std\left(P\right)}$$ (13)

Where *P*, *R*, *ET*, *SW* and *T* are the precipitation, land surface runoff, total evaporation, soil water content and temperature, respectively. *Ɛ* is the residual error term. *β*_*1*_, *β*_*2*,_ *β*_*3*_ and *β*_*4*_ are the regression coefficient of each driving factor. *Stc_R* is the standardized coefficient of runoff (Eq 13). Similarly, we can obtain the standardized coefficients of total evaporation (*Stc_ET*), soil water content (*Stc_SW*) and temperature (*Stc_T*).

The higher the value of standardized coefficient of a driving factor it is, the more large the effects of this factor will be[36]. Thereby, we used the Eq 14 to estimate the relative contribution of runoff (*C_R*) on the future precipitation changes. Similarly, we can obtain the contributions of total evaporation (*C_ET*), soil water content (*C_SW*) and temperature (*C_T*).


$$C\_R=\frac{\left|Stc\_R\right|}{\left|Stc\_R\right|+\left|Stc\_ET\right|+\left|Stc\_SW\right|+\left|Stc\_T\right|}$$ (14)

### Definition and calculation of annual extreme light rain days

Extreme light rainfall days here are defined as the days with rainfall (including days without rainfall) lower than the long-term 10th percentile. Based on the outputs of the daily precipitation during 2015–2100 from 12 CMIP6 models, we estimated the annual extreme light rainfall days in each grid. The mean value of the annual extreme light rainfall days in all terrestrial grids is regarded as the average number of annual drought days in North America.

## Results and discussion

### Mechanisms of emergent constraint technique

The basic idea of emergent constraint technique is to identify a variable *x* of the observable climate that both varies significantly across an ESM ensemble and that exhibits a statistically significant relationship with variations in some relevant variable *y* (i.e., Future annual precipitation growth rates in this study) describing the ESM’s future simulated climate state [37] (See schematic diagram in S1 Fig). The observed variable *x* normally has smaller uncertainties compared to the range of simulated values (S1 Fig) [16]. Thereby, by projecting the observed estimate of the variable *x* with its uncertainty onto the *y*-axis using an empirical linear relationship, we can obtain a more reliable and accurate variable *y* with smaller difference across models (See details in the caption of S1 Fig).

**Fig 1:**
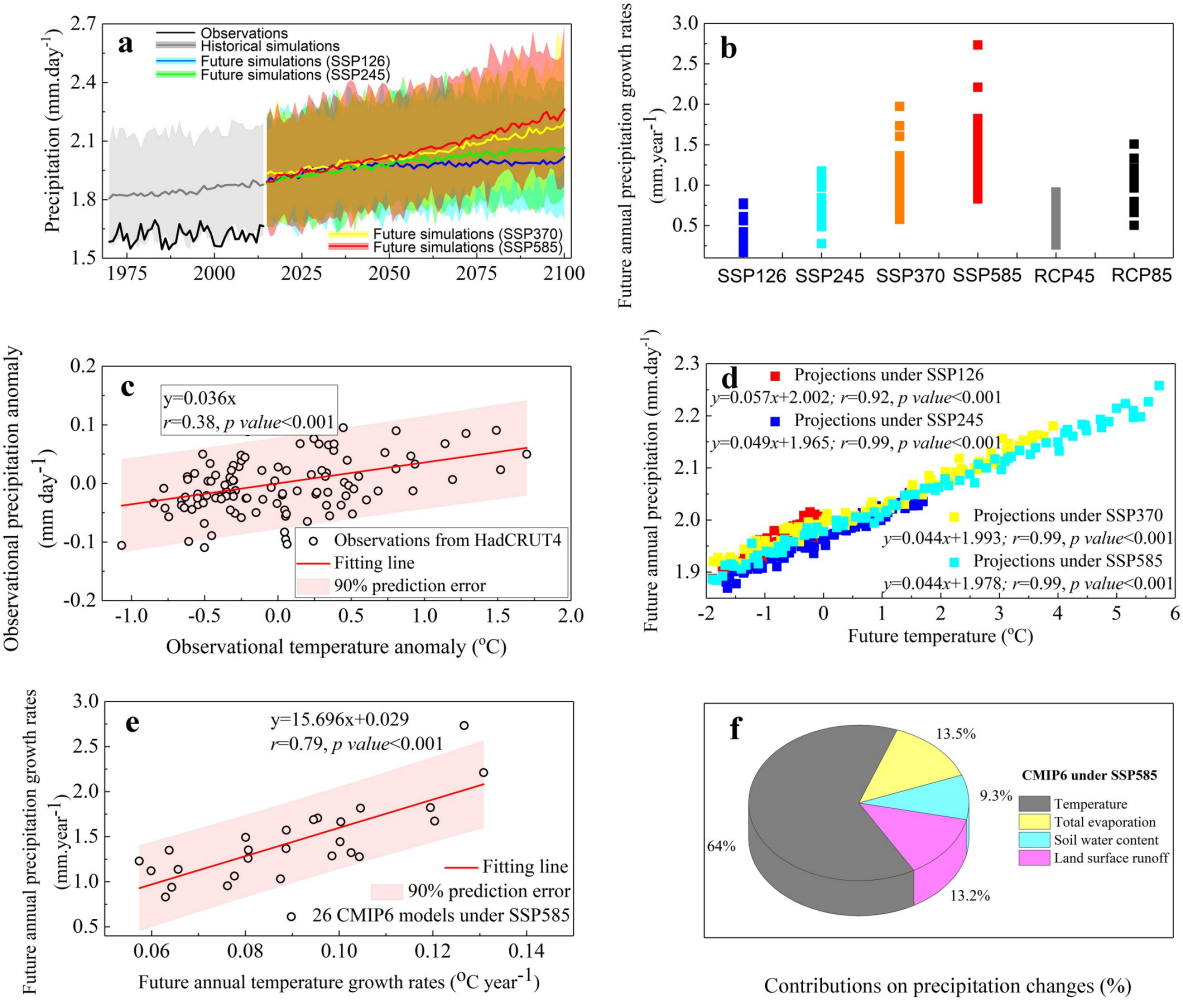
Future annual precipitation growth rate and its potential driving factors in North America. (**a**) presents the simulated annual average daily precipitation (mm day^-1^) from 27 CMIP6 models (1970–2100) and the observed precipitation from HadCRUT4 data set (1970–2014) (**b**) presents the large difference of predicted future annual precipitation growth rates (mm year^-1^) across the 27 CMIP6 models (See name of each model in S1 Table) under the emission scenarios of SSP126, SSP245, SSP370 and SSP585 and across the 33 CMIP5 models (See name of each model in S2 Table) under the emission scenarios of RCP45 and RCP85. (**c**) is the linear regression relationships between the observational temperature anomaly and the observed precipitation anomaly for the period of 1970–2014 from the HadCRUT4 data set, following the least-squares criterion. Each circle represents a year. (**d**) presents the linear relationships between future temperature and future annual precipitation for the CMIP6 models under the emission scenarios of SSP126, SSP245, SSP370, and SSP585 during 2015–2100. Each circle represents a year. (**e**) presents the linear relationships between the future annual growth rates in precipitation and in temperature for the CMIP6 models under SSP585 during 2015–2100. Each dot represents a model. (**f**) presents the relative contributions of temperature, total evaporation, soil water content and land surface runoff on the future precipitation changes in North America for the CMIP6 ensemble under SSP585 during 2015–2100. The geographical distribution of study area can be referred to Roque-Malo et al [38].

In North America, although the 27 CMIP6 models and the 33 CMIP5 models all indicate a trend of increasing precipitation during 2015–2100 (Figs 1a, 1b and S2), the predicted trend values have large differences across the models. We estimated that the future annual precipitation growth rates (i.e., future climate variable *y* in S1 Fig) from CMIP6 models are predicted to reach 0.3538 ± 0.1676 mm.year^-1^ (SSP126), 0.7043 ± 0.2182 mm.year^-1^ (SSP245), 1.0752 ± 0.3470 mm.year^-1^ (SSP370), and 1.4364 ± 0.4165 mm.year^-1^ (SSP585). Accordingly, the ranges across the CMIP5 models are also large (0.5248 ± 0.1825 mm.year^-1^ under RCP45 and 1.0139 ± 0.2393 mm.year^-1^ under RCP85). Before using the emergent constraint technique to reduce the uncertainties, we need to explore the dominant factor (i.e., Climate veriable *x* in S1 Fig) that drives the large inter-model spread of future precipitation changes.

The Clausius-Clapeyron equation (C–C, Eqs 7 and 8) indicates that a warming climate can have an increase of about 7% K^-1^ in the atmospheric water-holding capacity. Thereby, increases in the water vapor holding capacity of the atmosphere due to global warming largely attributed to the future increases in precipitation [39,40]. Assuming an unchanged atmospheric circulation, the thermodynamic equations (Eqs 9 and 10) from Kjellsson [28] also present a similar linear relationship between precipitation and temperature. That is, a degrees K increase in temperature will lead to 7% increase in precipitation [28]. Certainly, by considering radiative balance, positive sensitivity of precipitation to temperature will be also constrained by radiative cooling [41], and thus the increase rates in precipitation will be weakened by 4–6% K^-1^ [41,42], but generally precipitation still present a positive feedback behavior to temperature (1–3% K^-1^).

Supporting by this mechanism, the warming temperature in North America is identified as an important driving factor on the local precipitation increases, which is also verified by tight observational relationships (*r* = 0.38, *p-value* <0.001) between temperature and precipitation anomalies during 1901–2014 (Figs 1c and S3). The predictions from CMIP6 models also indicate a positive precipitation feedback to temperature changes under all the four emission scenarios (Fig 1d, *r*≥0.92, *p-value* <0.001), with the strong correlations (*p-value* <0.001) over most of North America’s land surface (covering 68.7–83.6% of the totals), which also confirms a strong constraint of temperature on local precipitation change.

However, the North American climate is also affected by other climate factors (e.g., monsoon, El Niño Southern Oscillation and the Arctic Oscillation) that can lead to extreme temperatures and precipitation [43–45]. These abnormal values can non-linearly affect trends in the climate of North America and possible cause us to obtain spurious precipitation–temperature relations. To exclude the interference of extreme climates, we smooth out extreme fluctuations using a series of moving windows with different lengths (5–10 years). This improves the reliability of the identified positive feedback behavior of precipitation to temperature [19,22] and indicates even tighter positive relationships between precipitation and temperature both in observations and simulations with the correlation coefficient increasing from 0.38 to 0.6–0.65 (S4 Fig). Furthermore, positive feedback behavior has been enhanced due to the increased sensitivity of precipitation to temperature (i.e., slope values in S4 Fig). This indicates that the dominant role of temperature in driving the local precipitation changes is robust, and becomes even more distinct after accounting for the disturbance other climate conditions.

The land water balance (Eq 11) indicates that precipitation changes may also be affected by other potential factors such as changes in total evaporation, soil moisture and land surface runoff [46–48]. Using a multiple regression (See Methods), we estimated the relative contributions of these drivers on the future precipitation changes. The results still indicate a primary role of temperature in affecting future precipitation changes persists under all the four SSPs, with the contributions of 47.6–80.8% (Figs 1f and S5, depends on the SSPs). By contrast, total evaporation (12.1–28.3%), soil moisture (3.4–20.8%) and land surface runoff (3.3–16.4%) all have much smaller contributions.

The linear relationships between annual growths in precipitation and in temperature (Figs 1e, S6 and S7, each circle represents a CMIP6/CMIP5 model) is tight for all historical periods or all future period under all the emission scenarios (0.60≤*r*≤0.88, p-valu*e*<0.001). These positive relationships indicate that the precipitation increases proportionally to the warming trends in North America. In other words, a warmer model (higher temperature) tens to exhibit a stronger precipitation increase, while a model with less warming tends to has a less strong precipitation increases. Thus, as long as the temperature increases, a stronger feedback implies more precipitation in both the past and the future. Thereby, due to the key role of warming on precipitation changes in North America, simulated historical annual temperature growth rate, we consider it the variable *x* for building the emergent constraint relationship with future annual precipitation growth rates (S1 Fig).

### Emergent constraint on future annual precipitation growth rates

During 1970–2014, the multi-model CMIP6 ensemble mean estimate of the annual precipitation growth rate is 0.58 ± 0.042 mm.year^-1^ per year. Based on the four observational datasets of HadCRUT4, NOAA, GISS and GHCN, the observed precipitation growth rate is 0.11 ± 0.17 mm.year^-1^ per year. We conclude that the CMIP6 models largely overestimate the historical precipitation increase trend in North America.

**Fig 2:**
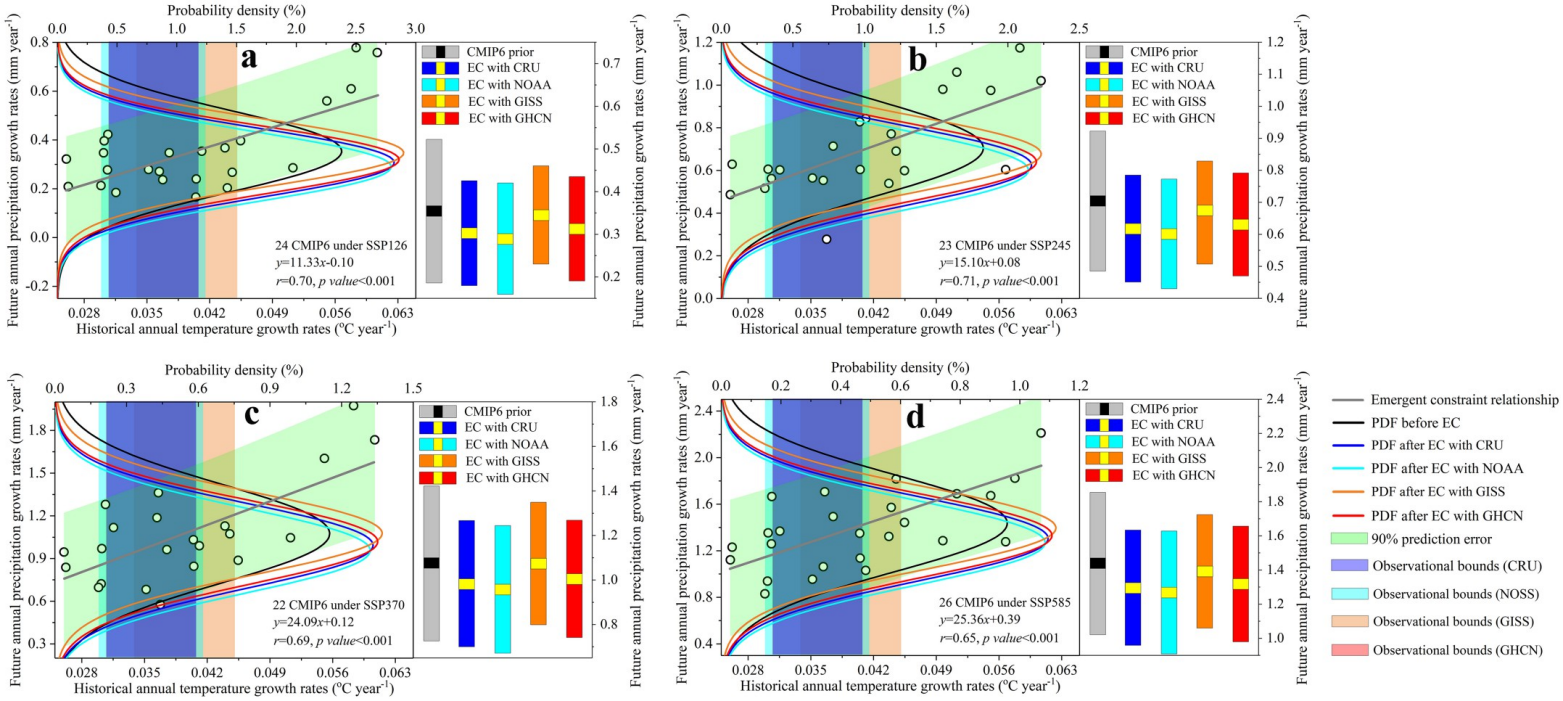
Emergent constraint (EC) on the future annual precipitation growth rates (2015–2100) in North America combining the observed annual temperature growth rates (1970–2014) for the CMIP6 projections. (**a**), (**b**), (**c**), and (**d**) are the emergent constraint relationships between the simulated historical annual temperature growth rates and the predicted future annual precipitation growth rates across the CMIP6 models under the emission scenarios of SSP126, SSP245, SSP370, and SSP585, respectively. Green shading is the 90% prediction error of the linear regression. Each dot represents a model. Four vertical shadings are the observed annual temperature growth rates (Mean ± one standard deviation) from HadCRUT4 (0.0357 ± 0.0050 °C year^-1^), NOAA (0.0346 ± 0.0048 °C year^-1^), GISS (0.0394 ± 0.0056 °C year^-1^) and GHCN (0.0365 ± 0.0050 °C year^-1^) data sets, respectively. Black curves and colorized curves are the PDFs of the future annual precipitation growth rates before and after the emergent constraint, respectively. Gray histograms and colorized histograms in the right panels of Fig 2 the constrained and unconstrained future annual precipitation growth rates (Mean ± one standard deviation), respectively.

By building linear relationships between annual growth of simulated historical temperature and that of future precipitation across the 27 CMIP6 models (Fig 2, p value<0.001), we found the strong correlations between them under all emission scenarios (i.e., SSP126, SSP245, SSP370 and SSP585). After cross-checking using the 33 CMIP5 models’ simulations, the tight constraint relationships still hold under both the emission scenarios of RCP45 and RCP85 (S8 Fig), implying the reliability of our introduced emergent constraint. These empirical relationships can reduce the uncertainties of the predicted future annual precipitation changes in North America by combining it with observations. Due to the large discrepancy of the observations across different data sets, the observed temperature from the four data sets (i.e., HadCRUT4, NOAA, GISS and GHCN, See vertical shadings in left panels of Fig 2) are collected for the same constraint processes for obtaining the reliable constrained results.

After the observed constraint, we find that the probability density functions (PDFs) of the constrained future annual precipitation growth rates have been shifted to the lower values under all the four SSPs (Colorized curves in Fig 2), in relative to the raw CMIP6 predictions (Black curves). The CMIP6 models originally predicted that the best estimates of future annual precipitation growth rates (Gray histograms in right panels of Fig 2) are 0.3538 mm.year^-1^ (SSP126), 0.7043 mm.year^-1^ (SSP245), 1.0752 mm.year^-1^ (SSP370), and 1.4364 mm.year^-1^ (SSP585). The constrained results indicate that the best estimates are more likely to reach 0.2903–0.3455 mm.year^-1^ (Depends on observational data sets), 0.6012–0.6748 mm.year^-1^, 0.9569–1.0742 mm.year^-1^, and 1.2691–1.3926 mm.year^-1^ respectively (Colorized histograms in right panels of Fig 2 and S6 Table). This shifting presents a largely overestimated future annual precipitation growth rates in North America by the original CMIP6 models’ predictions. The overestimated percentages of future water availability are 2.3–17.9% (SSP126), 4.2–14.6% (SSP245), 0.1–11.0% (SSP370) and 3.0–11.6% (SSP585).

In contrast, the raw 33 CMIP5 models’ predictions have underestimated the future terrestrial water supply from precipitation. After the constraint, the future annual precipitation growth rates have been increased from the raw predictions of 0.5248 mm.year^-1^ (RCP45) and 1.0139 mm.year^-1^ (RCP85) to the constrained values of 0.5445–0.5959 mm.year^-1^ and 1.0661–1.1533 mm.year^-1^(Depends on observational data sets), respectively (S8 Fig and S7 Table). Thereby, the CMIP5 models have underestimated future precipitation increases (3.8–13.7%), which is contrary to the constrained results of the CMIP6 models[49]. The main reason of this difference might be due to a stronger positive cloud feedback from decreasing extratropical low cloud coverage and albedo in CMIP6 models that tends to enhance the responses of temperature to increasing atmospheric CO_2_ concentration and then leads to an overestimated predictions of future precipitation increases.

No matter which observed data set we use, the PDFs of the constrained results (Colorized curves in Figs 2 and S8) all have been shifted tightly for each emission scenario in comparison with the raw CMIP5 and CMIP6 predictions (Black curves). The original standard deviations of future annual precipitation growth rates from CMIP6 models (Gray histograms in right panels of Fig 2) are 0.1676 mm.year^-1^ (SSP126), 0.2182 mm.year^-1^ (SSP126), 0.3470 mm.year^-1^ (SSP126) and 0.4165 mm.year^-1^ (SSP126). After the constraint, the standard deviations (Colorized histograms in right panels of Fig 2) have been successfully decreased to 0.1155–0.1303 mm.year^-1^, 0.1548–0.1712 mm.year^-1^, 0.2742–0.2869 mm.year^-1^ and 0.3326–0.3591 mm.year^-1^ respectively. Accordingly, the percentages of the reduced uncertainties are up to 13.8–31.1% after the constraint, indicating a more reliable and accurate estimate of future annual precipitation growth rates in relative the raw CMIP6 predictions. Consistently, the uncertainties of predictions from the 33 CMIP5 models are also reduced by 10.9–24.1% after constraining by the observed warming trends (S8 Fig).

Due to complex atmosphere–vegetation–soil interactions, positive feedback behavior of precipitation to temperature (Fig 1e and 1f) are unexpectedly nonexistent in the southern regions of North America (10.6–14.4% of total areas of North America with negative correlations), i.e., an increased temperature but with a decreased precipitation. In these regions, the mechanisms underpin the emergent constraint relationships might be unrealistic, due to dominant role of other factors in driving the local precipitation changes, instead of warming temperature. After excluding these regions, the emergent constraint relationships (red fitting lines in S8 Fig) basically remained the same under all the SSPs in comparison with the constraint relationships that include the southern North America (Black lines in S9 Fig and Gray lines in Fig 2), indicating a slight influence of such regions on the area-averaged emergent constraint relationships.

### Implications on future warming trends

**Fig 3:**
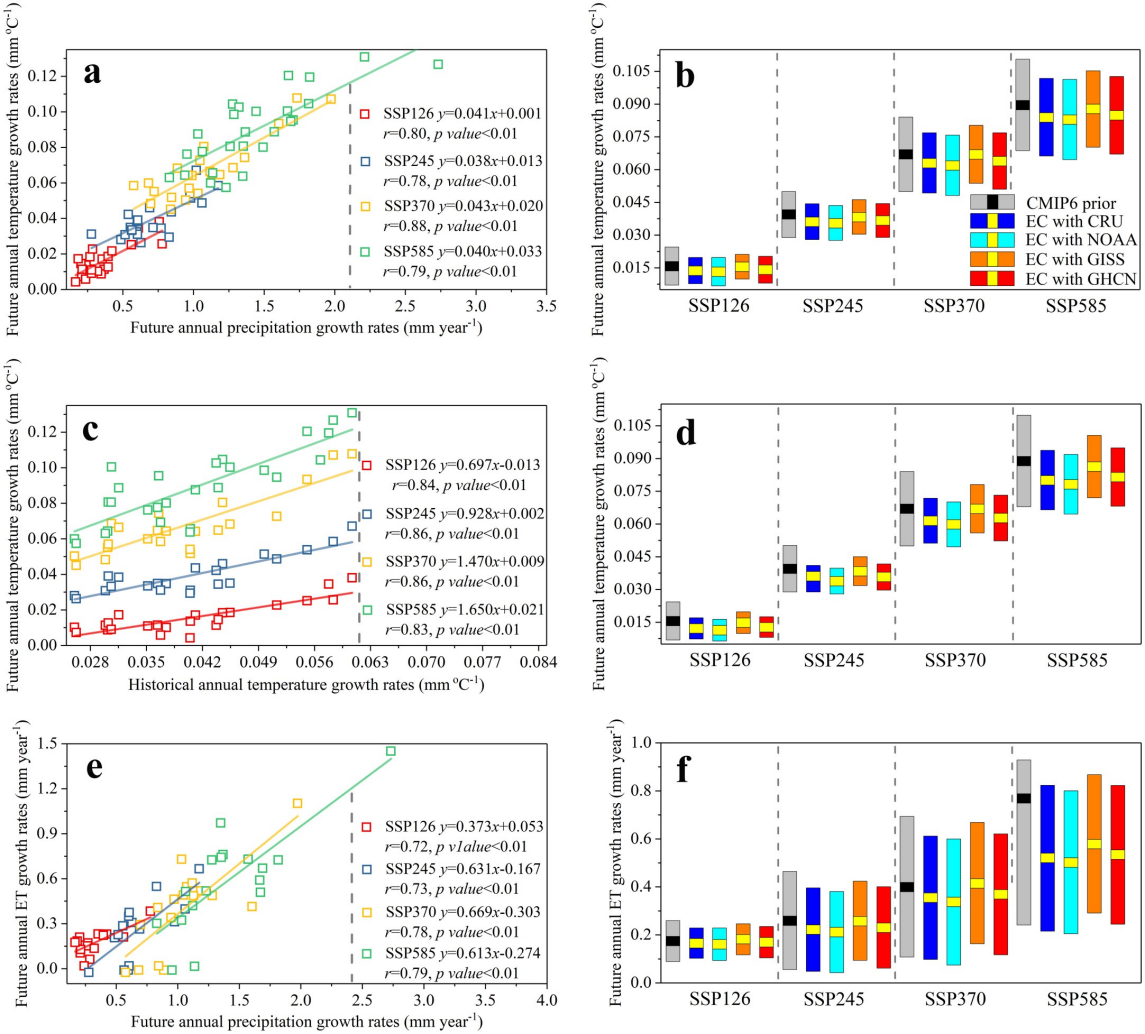
Emergent constraints on the future annual growth rates of temperature and that of total evaporation (ET) based on the CMIP6 predictions during 2015–2100. (**a**) presents the linear relationships between future annual growth rates of precipitation and that of temperature. (**b**) presents the constrained and the unconstrained future annual temperature growth rates by applying the constrained future annual precipitation growth rates and the relationships in Fig 3a. (**c**) presents the linear relationships between annual growth rates of historical temperature (1970–2014) and that of future precipitation (2015–2100). (**d**) presents the constrained and the unconstrained future annual temperature growth rates by applying the observed warming trends and the relationships in Fig 3c. (**e**) presents the linear relationships between future annual growth rates of precipitation and that of total evaporation. (**f**) presents the constrained and the unconstrained future annual total evaporation growth rates by applying the constrained future annual precipitation growth rates and the relationships in Fig 3e.

Future annual growth rates of temperature and that of precipitation exist a great positive linear relationship between them across the 27 CMIP6 models under all the four SSPs (Fig 3a, Each circle represents a model), i.e., a model with a less precipitation normally associates with a colder temperature. In Fig 2, we obtained the more reliable and accurate estimates of future annual precipitation growth rates after the constraint, which may bring some implications on future temperature changes in North America. By projecting the constrained future annual precipitation growth rates (Obtaining from Fig 2) into *y*-axis through the significant statistical relationships of Fig 3a, then we can obtain the constrained future warming trends.

The constrained results reveal an overestimated future warming trends in North America. The raw CMIP6 outputs (Gray histograms in Fig 3b) predict an increasing temperature of 0.0158 ± 0.0086 °C year^-1^ (SSP126), 0.0395 ± 0.0105 °C year^-1^ (SSP245), 0.0671 ± 0.0171 °C year^-1^ (SSP370) and 0.0897 ± 0.0210 °C year^-1^ (SSP585). After the constraint (Colorized histograms in Fig 3b and S8 Table), we estimated that the rates of annual temperature increases have been decreased to 0.0132–0.0155 ± 0.0057–0.0065 °C year^-1^,0.0356–0.0383 ± 0.0078–0.0082°C year^-1^, 0.0619–0.0670 ± 0.0130–0.0138 °C year^-1^ and 0.0830–0.0879 ± 0.0175–0.0185 °C year^-1^, respectively. This shifting indicates that the raw CMIP6 predictions overestimated the future temperature increases in North America under all the four SSPs, with the overvalued percentages up to 0.1 ± 16.5%. On the contrary, the future warming trends have been underestimated by the CMIP5 models (2.8 ± 11.9%, S10 Fig and S9 Table).

To verify the reliability of the constrained results of future warming trends in Fig 3b, we introduced a new emergent constraint that relies on the significant statistical relationship between annual growth rates in historical temperature and that of future temperature (Fig 1c). The plausible mechanisms underpin the relationships are that the responses of temperature are proportional to the radiative forcing, and thus, as long as the forcing increases, stronger feedbacks imply higher temperature in both the past and the future [50,51]. After constraining by the observed temperature from the four different data sets, we found that the future warming trends in North America have been overestimated by the raw CMIP6 predictions under all the SSPs (overvalued by 0.1 ± 26.9%, Fig 3d and S10 Table), while the original CMIP5 outputs underestimated future temperature increases (3.3 ± 15.6%, S10 Fig). This result (Fig 3c and 3d) is highly in agreement with the conclusions that are obtained by applying the constrained future precipitation growth rates (Fig 3a and 3b). In particular, the uncertainties of the temperature predictions have been successfully reduced by 31.6–46.0% (CMIP6) and 13.0–24.1% (CMIP5) due to the narrowed standard deviations (Fig 3d), implying a more reliable and accurate estimate of future temperature changes.

### Implications on future total evaporation

Precipitation changes can alter evaporation [52,53]. These potential effects are supported by the strong positive linear relationships between future annual growth rates of total evaporation and that of precipitation across the CMIP6 models (Fig 3e, i.e., a model with a higher precipitation normally has a higher total evaporation). After projecting the constrained future annual growth rates (Obtaining from Fig 2) into *y*-axis through the relationships in Fig 3e, we estimated that the future annual total evaporation growth rates (Colorized histograms in Fig 3f and S12 Table) are more likely to reach 0.1613–0.1819 ± 0.0640–0.0684 mm.year^-1^ (SSP126), 0.2121–0.2586 ± 0.1659–0.1744 mm.year^-1^ (SSP245), 0.3375–0.4160 ± 0.2519–0.2623 mm.year^-1^ (SSP370) and 0.5033–0.5789 ± 0.2890–0.3043 mm.year^-1^ (SSP585). In comparison with the raw CMIP6 predictions (Gray histograms in Fig 3f), the constrained results reveal that the future annual total evaporation growth rates have been overestimated by -4.4–18.5% under all the four SSPs, due to the overestimated future precipitation increases. On the contrary, the original CMIP5 outputs underestimated the future water losses from land surface to atmosphere (1.0–14.2%, S13 Table).

“Precipitation minus evapotranspiration” here is used to metric the water availability over land surface [54–56]. Based on the constrained CMIP6 predictions of precipitation and total evaporation (Colorized histograms in Figs 2 and 3f), we estimated the constrained future water availability changes (S14 Table). In relative to the raw CMIP6 outputs, the future water availability growth rates have been largely overestimated by 2.4–28.2% (S14 Table) due to the largely overvalued precipitation increases (Fig 2). This is also supported by investigating the negative relationships (S12 Table) between the future annual precipitation growth rates and the future yearly changes in average annual extreme light rainfall days (See Method). These negative relationships indicate that a model with a fewer precipitation normally has a higher frequency of average annual extreme light rainfall days. Thus, a potential overestimated future annual precipitation growth rates in CMIP6 models represents an underestimated frequency in future average annual extreme light days.

## Conclusions

In this study, we successfully captured the emergent constraint relationships between annual growth rates of simulated historical temperature and that of future precipitation in North America, across 27 CMIP6 models (underSSP126, SSP245, SSP370 and SSP585) and 33 CMIP5 models (under RCP45 and RCP85). Combining the observed temperature from four different data sets, the emergent constraint relationships successfully reduced the uncertainties of precipitation predictions by 13.8–31.1%, and also reveal an overestimation of future precipitation growth rates under all the four SSPs by the raw CMIP6 predictions. Furthermore, the constrained future precipitation changes are further applied to capture the more accurate future temperature trends, which indicate that the future annual temperature growth rates have been overestimated by the CMIP6 models (overestimated by 0.1 ± 16.5% by the originally predicted), while the CMIP5 models underestimated the future warming trends by around 2.8 ± 11.9%.

## Supporting information

S1 Fig Schematic diagram of emergent constraint technique. Note: Each circle represents a model. Red fitting line is the emergent constraint relationship between “simulated historical climate variable *x* (i.e., historical annual temperature growth rates in Fig 2)” and “predicted future climate variable *y* (i.e., future annual precipitation growth rates)”. Baby blue curve and gray curve are the probability density functions (PDFs) of the observed climate variable *x* and simulated historical climate variable *x*. Clearly, the observed climate variable *x* has less uncertainties in compared to the range of simulated values of climate variable *x*. Thereby, by projecting the observed climate variable *x* into *y*-axis through the emergent constraint relationship, we can obtain the more accurate future future climate variable *y* with less uncertainties in relative the raw models’ predictions. Dark blue curve and dark curve are the PDFs of the constrained and the unconstrained future climate variable *y*.
(TIF)

S2 Fig Simulated annual average daily precipitation (mm day^-1^) from the 33 CMIP5 models (1970–2100) and the observed precipitation from HadCRUT4 data set (1970–2005). See name of each model in S2 Table.
(TIF)

S3 Fig Linear regression relationships between the observational temperature anomaly and the observed precipitation anomaly for the period of 1970–2014. (**a**), (**b**) and (**c**) are relations for the data from NOAA, GHCN, and GISS, respectively.
(TIF)

S4 Fig Linear relationships between future annual precipitation growth rates and future annual temperature growth rates in North America. Each circle represents a CMIP6 model. (**a**), (**b**) and (**c**) are the linear relations for the CMIP6 models under SSP126, SSP245, and SSP370, respectively.
(TIF)

S5 Fig Linear relationships between future annual precipitation growth rates and future annual temperature growth rates in North America. Each circle represents a CMIP5 model. (**a**) and (**b**) are the linear relations for the CMIP5 models under RCP45 and RCP85, respectively.
(TIF)

S6 Fig Linear relationships between observed temperature anomaly and observed precipitation anomaly using moving windows with different lengths (5–10 years). At first, we calculated temperature/precipitation using moving windows with different lengths (5–years) during 1982–2015. Then, the calculated values are used to build the linear relationships. For instance,(**a**) presents the linear relationship between observed temperature anomaly and observed precipitation anomaly using moving windows with the length of 5 years. Blue histograms in (**b**) and (**c**) are the correlation coefficient and the slope values (i.e., sensitivity of precipitation to temperature) respectively, with different lengths of moving windows (5–years). Gray histogram is the values without moving windows (i.e., the values in Fig 1c of the Main Text).
(TIF)

S7 Fig Relative contributions of temperature, total evaporation, soil water content and land surface runoff on the future precipitation changes in North America for the CMIP6 ensemble during 2015–2100. (**a**), (**b**) and (**c**) are the contributions for the emission scenarios of SSP126, SSP245, and SSP370, respectively.
(TIF)

S8 Fig Emergent constraint (EC) on the future annual precipitation growth rates (2006–2100) in North America combining the observed annual temperature growth rates (1970–2005) for the CMIP5 projections. (**a**) and (**b**) are the emergent constraint relationships between the simulated historical annual temperature growth rates and the predicted future annual precipitation growth rates across the CMIP5 models under the emission scenarios of RCP45 and RCP85, respectively. Green shading is the 90% prediction error of the linear regression. Each dot represents a model. Four vertical shadings are the observed annual temperature growth rates (Mean ± one standard deviation) from HadCRUT4, NOAA, GISS and GHCN data sets, respectively. Black curves and colorized curves are the PDFs of the future annual precipitation growth rates before and after the emergent constraint, respectively. Gray histograms and colorized histograms in the right panels of S9 Fig the constrained and unconstrained future annual precipitation growth rates (Mean ± one standard deviation), respectively.
(TIF)

S9 Fig Emergent constraint relationships between simulated historical annual temperature growth rates and predicted future annual precipitation growth rates across the CMIP6 models. Each dot represents a model. (**a**), (**b**), (**c**) and (**d**) are the constraint relationships under SSP126, SSP245, SSP370 and SSP585, respectively. Red fitting lines are the emergent constraint relationships after excluding the regions with negative correlations between future precipitation and future temperature (See locations of negative regions in Fig 1d of the Main Text). Dark fitting lines are the emergent constraint relationships including these regions (i.e., the linear relationships in Fig 2 of the Main Text).
(TIF)

S10 Fig Emergent constraints on the future annual growth rates of temperature and that of total evaporation (ET) based on the CMIP5 predictions under emission scenarios of RCP45 and RCP85 during 2006–2100. (**a**) presents the linear relationships between future annual growth rates of precipitation and that of temperature. (**b**) presents the constrained and the unconstrained future annual temperature growth rates by applying the constrained future annual precipitation growth rates. (**c**) presents the linear relationships between annual growth rates of historical temperature (1970–2014) and that of future precipitation (2015–2100). (**d**) presents the constrained and the unconstrained future annual temperature growth rates by applying the observed warming trends. (**e**) presents the linear relationships between future annual growth rates of precipitation and that of total evaporation. (**f**) presents the constrained and the unconstrained future annual total evaporation growth rates by applying the constrained future annual precipitation growth rates.
(TIF)

S11 Fig Relationships between the future annual precipitation growth rates and the future yearly changes in average annual light rainfall days based on CMIP6 predictions. (**a**), (**b**), (**c**) and (**d**) are the relationships under SSP126, SSP245, SSP370 and SSP585, respectively. Each Circle represents a CMIP6 model.
(TIF)

S1 Table Full name of the CMIP6 models for collecting the monthly data of land surface temperature and precipitation during 1970–2100. (DOCX)

S2 Table Full name of the CMIP5 models for collecting the monthly data of land surface temperature and precipitation during 1970–2100. (DOCX)

S3 Table Full name of the CMIP6 models for collecting the monthly data of total evaporation under 2015–2100. (DOCX)

S4 Table Full name of the CMIP6 models for collecting the monthly data of soil water content during 2015–2100. (DOCX)

S5 Table Full name of the CMIP6 models for collecting the monthly data of land surface runoff during 1970–2100. (DOCX)

S6 Table Emergent constraint on the future annual precipitation growth rates in North America for the period of 2015–2100 based on CMIP6 projections. Overestimated future precipitation increase = │constrained precipitation–unconstrained precipitation│/unconstrained precipitation; Reduced uncertainty = │constrained standard deviation–unconstrained standard deviation│/unconstrained standard deviation.
(DOCX)

S7 Table Emergent constraint on the future annual precipitation growth rates in North America for the period of 2006–2100 based on CMIP5 projections. (DOCX)

S8 Table Constraint on the future annual temperature growth rates in North America for the period of 2015–2100 based on CMIP6 projections by using the constrained future annual precipitation growth rates. (DOCX)

S9 Table Constraint on the future annual temperature in North America for the period of 2015–2100 based on CMIP5 projections by using constrained future annual precipitation growth rates. (DOCX)

S10 Table Constraint on the future annual temperature growth rates in North America for the period of 2015–2100 based on CMIP6 projections by using the observed annual temperature growth rates. (DOCX)

S11 Table Constraint on the future annual temperature in North America for the period of 2015–2100 based on CMIP5 projections by using observed annual temperature growth rates. (DOCX)

S12 Table Constraint on the future annual total evaporation (ET) growth rates in North America for the period of 2015–2100 based on CMIP6 projections by using the constrained future annual precipitation growth rates. (DOCX)

S13 Table Constraint on the future annual total evaporation (ET) in North America for the period of 2015–2100 based on CMIP5 projections by using constrained future annual precipitation growth rates. (DOCX)

S14 Table Water availability based on the constrained precipitation and the constrained total evaporation. Note: The unconstrained water availability = the unconstrained precipitation–the unconstrained total evaporation; the constrained water availability = the constrained precipitation–the constrained total evaporation; Overestimated percentages = │constrained water availability–unconstrained water availability│/unconstrained water availability.
(DOCX)

S15 Table Full name of the CMIP6 models for collecting the daily data of precipitation during 2015–2100. (DOCX)

## References

[1] Seager R., Tzanova A., and Nakamura J., Drought in the Southeastern United States: Causes, Variability over the Last Millennium, and the Potential for Future Hydroclimate Change*. Journal of Climate, 2009. 22(19): p. 5021–5045.

[2] Kam J., Sheffield J., and Wood E.F., Changes in drought risk over the contiguous United States (1901–2012): The influence of the Pacific and Atlantic Oceans. Geophysical Research Letters, 2014. 41(16): p. 5897–5903.

[3] Grigg N.S., The 2011–2012 drought in the United States: new lessons from a record event. International Journal of Water Resources Development, 2014. 30(2): p. 183–199.

[4] Chikamoto Y., et al., Multi-year predictability of climate, drought, and wildfire in southwestern North America. Sci Rep, 2017. 7(1): p. 6568. doi: 10.1038/s41598-017-06869-7 28747719 PMC5529505

[5] Littell J.S., et al., A review of the relationships between drought and forest fire in the United States. Global Change Biology, 2016. 22(7): p. 2353–2369. doi: 10.1111/gcb.13275 27090489

[6] Jeong D.I., Sushama L., and Naveed Khaliq M., The role of temperature in drought projections over North America. Climatic Change, 2014. 127(2): p. 289–303.

[7] Eyring V., et al., Overview of the Coupled Model Intercomparison Project Phase 6 (CMIP6) experimental design and organization. Geosci. Model Dev., 2016. 9(5): p. 1937–1958.

[8] Knutti R. and Sedláček J., Robustness and uncertainties in the new CMIP5 climate model projections. Nature Climate Change, 2013. 3(4): p. 369–373.

[9] Wang C., et al., A global perspective on CMIP5 climate model biases. Nature Climate Change, 2014. 4(3): p. 201–205.

[10] Allen R.J. and Luptowitz R., El Niño-like teleconnection increases California precipitation in response to warming. Nature Communications, 2017. 8(1): p. 16055. 10.1038/ncomms16055 PMC5504297 28681837

[11] Pachauri K. and Meyer A., CLIMATE CHANGE 2014. SYNTHESIS REPORT. Environmental Policy Collection, 2014. 27(2): p. 408.

[12] Haarsma R.J., et al., High Resolution Model Intercomparison Project (HighResMIP v1.0) for CMIP6. Geosci. Model Dev., 2016. 9(11): p. 4185–4208.

[13] Cook B.I., et al., Twenty-First Century Drought Projections in the CMIP6 Forcing Scenarios. Earth’s Future, 2020. 8(6).

[14] Beobide-Arsuaga G., et al., Uncertainty of ENSO-amplitude projections in CMIP5 and CMIP6 models. Climate Dynamics, 2021. 56(11–12): p. 3875–3888.

[15] Zhang S. and Chen J., Uncertainty in Projection of Climate Extremes: A Comparison of CMIP5 and CMIP6. Journal of Meteorological Research, 2021. 35(4): p. 646–662.

[16] Hall A., et al., Progressing emergent constraints on future climate change. Nature Climate Change, 2019. 9(4): p. 269–278.

[17] Fasullo J.T. and Trenberth K.E., A less cloudy future: the role of subtropical subsidence in climate sensitivity. Science, 2012. 338(6108): p. 792–4. doi: 10.1126/science.1227465 23139331

[18] Cox P.M., et al., Sensitivity of tropical carbon to climate change constrained by carbon dioxide variability. Nature, 2013. 494(7437): p. 341–344. doi: 10.1038/nature11882 23389447

[19] Chai Y., et al., Constrained CMIP6 projections indicate less warming and a slower increase in water availability across Asia. Nature Communications, 2022. 13(1): p. 4124. doi: 10.1038/s41467-022-31782-7 35840591 PMC9287300

[20] Sherwood S.C., Bony S., and Dufresne J.-L., Spread in model climate sensitivity traced to atmospheric convective mixing. Nature, 2014. 505(7481): p. 37–42. doi: 10.1038/nature12829 24380952

[21] Cox P.M., Huntingford C., and Williamson M.S., Emergent constraint on equilibrium climate sensitivity from global temperature variability. Nature, 2018. 553(7688): p. 319–322. doi: 10.1038/nature25450 29345639

[22] Chai Y., et al., Constraining Amazonian land surface temperature sensitivity to precipitation and the probability of forest dieback. npj Climate and Atmospheric Science, 2021. 4(1): p. 6.

[23] Chadburn S.E., et al., An observation-based constraint on permafrost loss as a function of global warming. Nature Climate Change, 2017. 7(5): p. 340–344.

[24] Zhu B., et al., Constrained tropical land temperature-precipitation sensitivity reveals decreasing evapotranspiration and faster vegetation greening in CMIP6 projections. npj Climate and Atmospheric Science, 2023. 6(1): p. 91.

[25] Kwiatkowski L., et al., Emergent constraints on projections of declining primary production in the tropical oceans. Nature Climate Change, 2017. 7(5): p. 355–358.

[26] Thackeray C.W. and Hall A., An emergent constraint on future Arctic sea-ice albedo feedback. Nature Climate Change, 2019. 9(12): p. 972–978.

[27] O’Gorman P.A., Sensitivity of tropical precipitation extremes to climate change. Nature Geoscience, 2012. 5(10): p. 697–700.

[28] Kjellsson J., Weakening of the global atmospheric circulation with global warming. Climate Dynamics, 2014. 45(3–4): p. 975–988.

[29] Held I.M. and Soden B.J., Robust Responses of the Hydrological Cycle to Global Warming. Journal of Climate, 2006. 19(21): p. 5686–5699.

[30] Hoogeveen J., et al., GlobWat–a global water balance model to assess water use in irrigated agriculture. Hydrol. Earth Syst. Sci., 2015. 19(9): p. 3829–3844.

[31] van Beek L.P.H., Wada Y., and Bierkens M.F.P., Global monthly water stress: 1. Water balance and water availability. Water Resources Research, 2011. 47(7).

[32] Thomas G. and Henderson-Sellers A., Global and continental water balance in a GCM. Climatic Change, 1992. 20(4): p. 251–276.

[33] Wang S., et al., Recent global decline of CO2 fertilization effects on vegetation photosynthesis. Science, 2020. 370(6522): p. 1295–1300. doi: 10.1126/science.abb7772 33303610

[34] Jung M., et al., Compensatory water effects link yearly global land CO2 sink changes to temperature. Nature, 2017. 541(7638): p. 516–520. doi: 10.1038/nature20780 28092919

[35] Humphrey V., et al., Soil moisture–atmosphere feedback dominates land carbon uptake variability. Nature, 2021. 592(7852): p. 65–69. doi: 10.1038/s41586-021-03325-5 33790442 PMC8012209

[36] Chai Y., et al., Homogenization and polarization of the seasonal water discharge of global rivers in response to climatic and anthropogenic effects. Science of The Total Environment, 2020. 709: p. 136062. doi: 10.1016/j.scitotenv.2019.136062 31887524

[37] Williamson M.S., et al., Emergent constraints on climate sensitivities. Reviews of Modern Physics, 2021. 93(2): p. 025004.

[38] Roque-Malo S. and Kumar P., Patterns of change in high frequency precipitation variability over North America. Scientific Reports, 2017. 7(1): p. 10853. doi: 10.1038/s41598-017-10827-8 28924195 PMC5603571

[39] Berg P., et al., Seasonal characteristics of the relationship between daily precipitation intensity and surface temperature. Journal of Geophysical Research, 2009. 114(D18).

[40] Chen G., et al., Testing the Clausius-Clapeyron constraint on the aerosol-induced changes in mean and extreme precipitation. Geophysical Research Letters, 2011. 38(4): p. n/a–n/a.

[41] Stephens G.L. and Hu Y., Are climate-related changes to the character of global-mean precipitation predictable? Environmental Research Letters, 2010. 5(2).

[42] Vecchi G.A. and Soden B.J., Global Warming and the Weakening of the Tropical Circulation. Journal of Climate, 2007. 20(17): p. 4316–4340.

[43] Lim Y.K., et al., The impact of SST-forced and unforced teleconnections on 2015/16 El Nino winter precipitation over the western United States. J Clim, 2018. 31(15): p. 5825–5844. 30197468 10.1175/JCLI-D-17-0218.1 PMC6121706

[44] Cai W., et al., Climate impacts of the El Niño–Southern Oscillation on South America. Nature Reviews Earth & Environment, 2020. 1(4): p. 215–231.

[45] Wang H. and Asefa T., Impact of different types of ENSO conditions on seasonal precipitation and streamflow in the Southeastern United States. International Journal of Climatology, 2018. 38(3): p. 1438–1451.

[46] Wei J., Su H., and Yang Z.-L., Impact of moisture flux convergence and soil moisture on precipitation: a case study for the southern United States with implications for the globe. Climate Dynamics, 2016. 46(1): p. 467–481.

[47] Tawfik A.B. and Steiner A.L., The role of soil ice in land-atmosphere coupling over the United States: A soil moisture–precipitation winter feedback mechanism. Journal of Geophysical Research, 2011. 116(D2).

[48] Tuttle S. and Salvucci G., Empirical evidence of contrasting soil moisture-precipitation feedbacks across the United States. Science, 2016. 352(6287): p. 825–8. doi: 10.1126/science.aaa7185 27174987

[49] Sheffield J., Barrett A., and Barrie D.B., *Regional climate processes and projections for North America*: *CMIP3/CMIP5 differences*, *attribution and outstanding issues*. 2014.

[50] Tokarska K.B., et al., Past warming trend constrains future warming in CMIP6 models. Science Advances. 6(12): p. eaaz9549. doi: 10.1126/sciadv.aaz9549 32206725 PMC7080456

[51] Jiménez-de-la-Cuesta D. and Mauritsen T., Emergent constraints on Earth’s transient and equilibrium response to doubled CO2 from post-1970s global warming. Nature Geoscience, 2019. 12(11): p. 902–905.

[52] Li D., Assessing the impact of interannual variability of precipitation and potential evaporation on evapotranspiration. Advances in Water Resources, 2014. 70: p. 1–11.

[53] Yuan W., et al., Impacts of precipitation seasonality and ecosystem types on evapotranspiration in the Yukon River Basin, Alaska. Water Resources Research, 2010. 46(2).

[54] Liu Y., et al., Response of evapotranspiration and water availability to the changing climate in Northern Eurasia. Climatic Change, 2014. 126(3): p. 413–427.

[55] Cramer M.D. and Hoffman M.T., The Consequences of Precipitation Seasonality for Mediterranean-Ecosystem Vegetation of South Africa. PLOS ONE, 2015. 10(12): p. e0144512. doi: 10.1371/journal.pone.0144512 26650081 PMC4674101

[56] Ying S. and Xue-Jie G., Projection of Water Availability in the Miyun Watershed from an RCM Simulation. Atmospheric and Oceanic Science Letters, 2012. 5(6): p. 468–472.

