# Supplementary figures and images for: Uncertainty reduction for precipitation prediction in North America
Source: PLoS One. 2024 May 22;19(5):e0301759. doi: 10.1371/journal.pone.0301759 (PMC11111050; doi:10.1371/journal.pone.0301759)

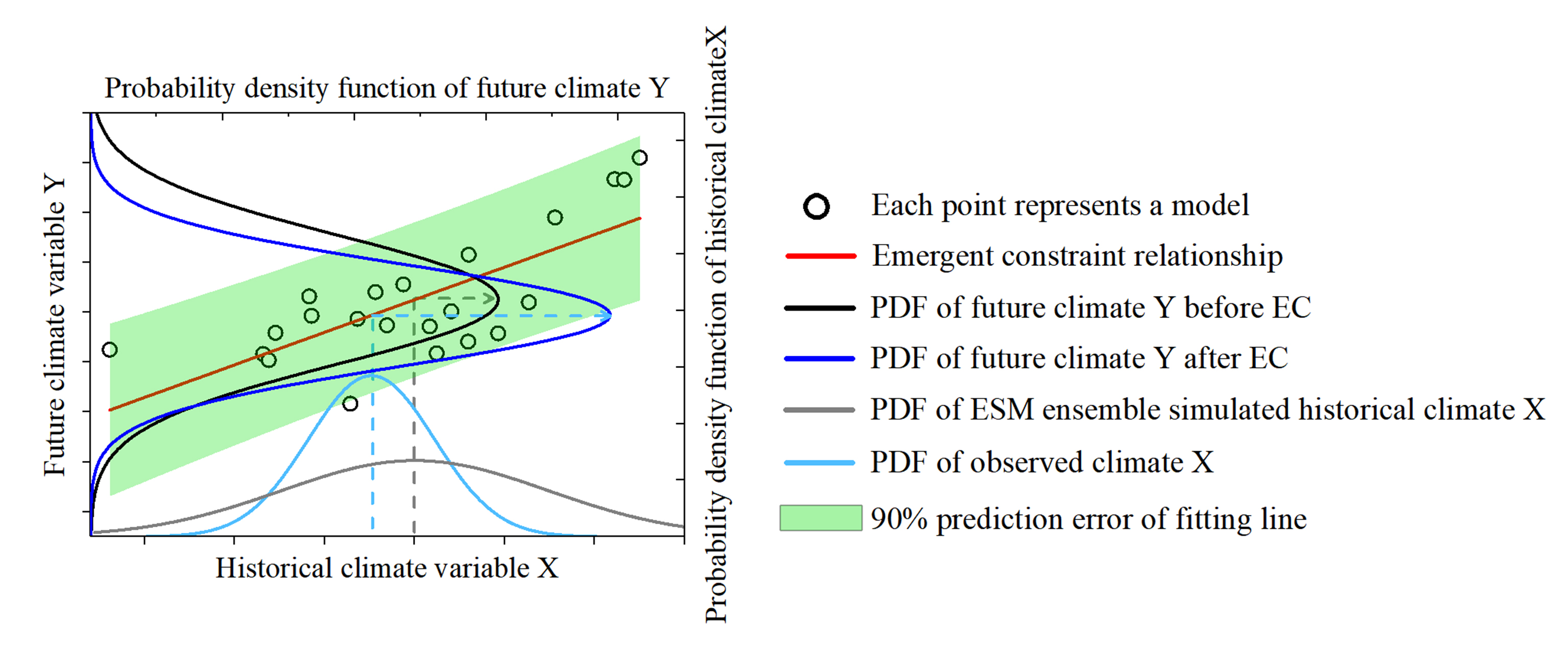

Supplement: S1 Fig — Note: Each circle represents a model. Red fitting line is the emergent constraint relationship between “simulated historical climate variable x (i.e., historical annual temperature growth rates in Fig 2)” and “predicted future climate variable y (i.e., future annual precipitation growth rates)”. Baby blue curve and gray curve are the probability density functions (PDFs) of the observed climate variable x and simulated historical climate variable x. Clearly, the observed climate variable x has less uncertainties in compared to the range of simulated values of climate variable x. Thereby, by projecting the observed climate variable x into y-axis through the emergent constraint relationship, we can obtain the more accurate future future climate variable y with less uncertainties in relative the raw models’ predictions. Dark blue curve and dark curve are the PDFs of the constrained and the unconstrained future climate variable y. (TIF) [file pone.0301759.s001.tif]

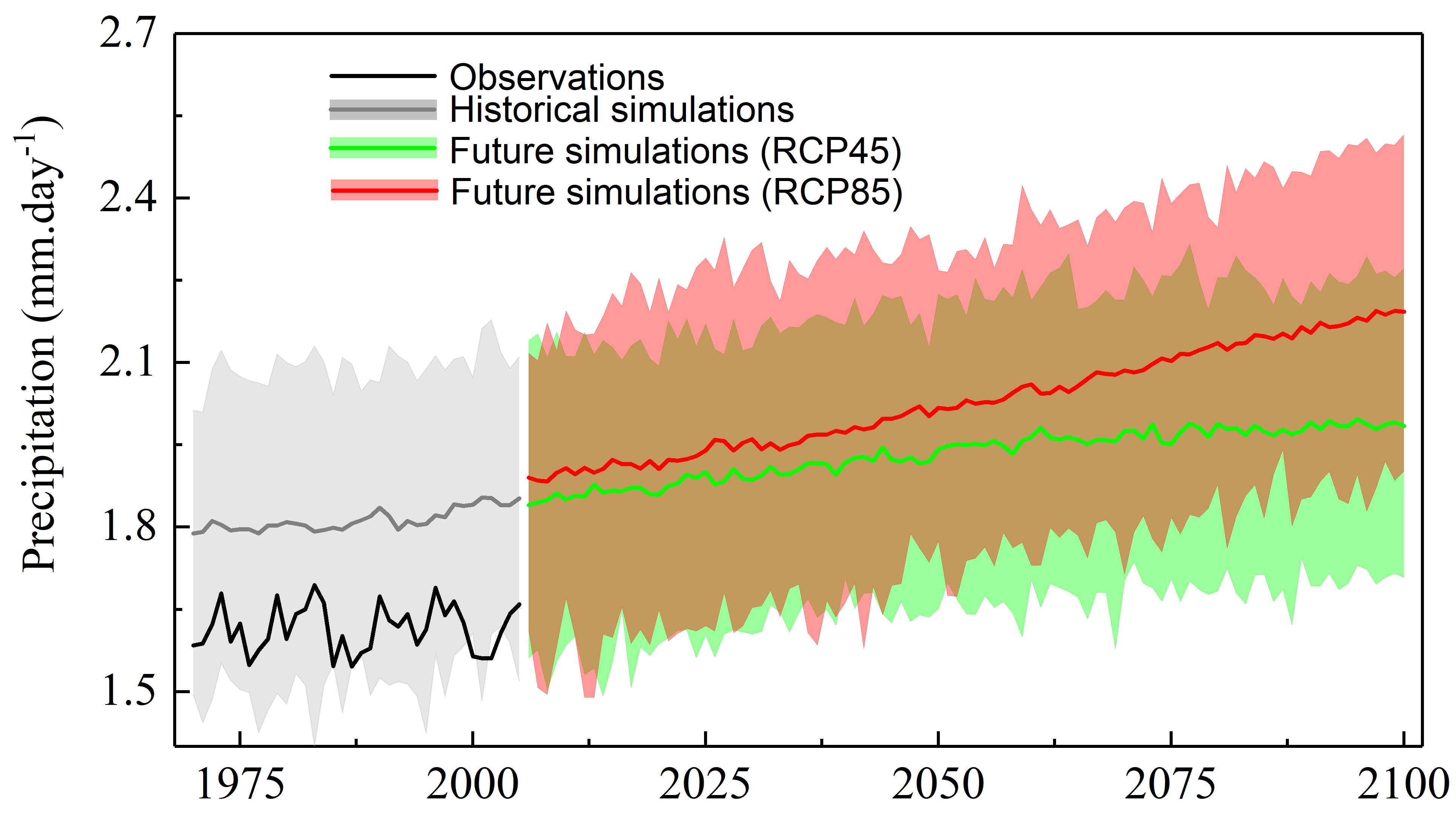

Supplement: S2 Fig — See name of each model in S2 Table. (TIF) [file pone.0301759.s002.tif]

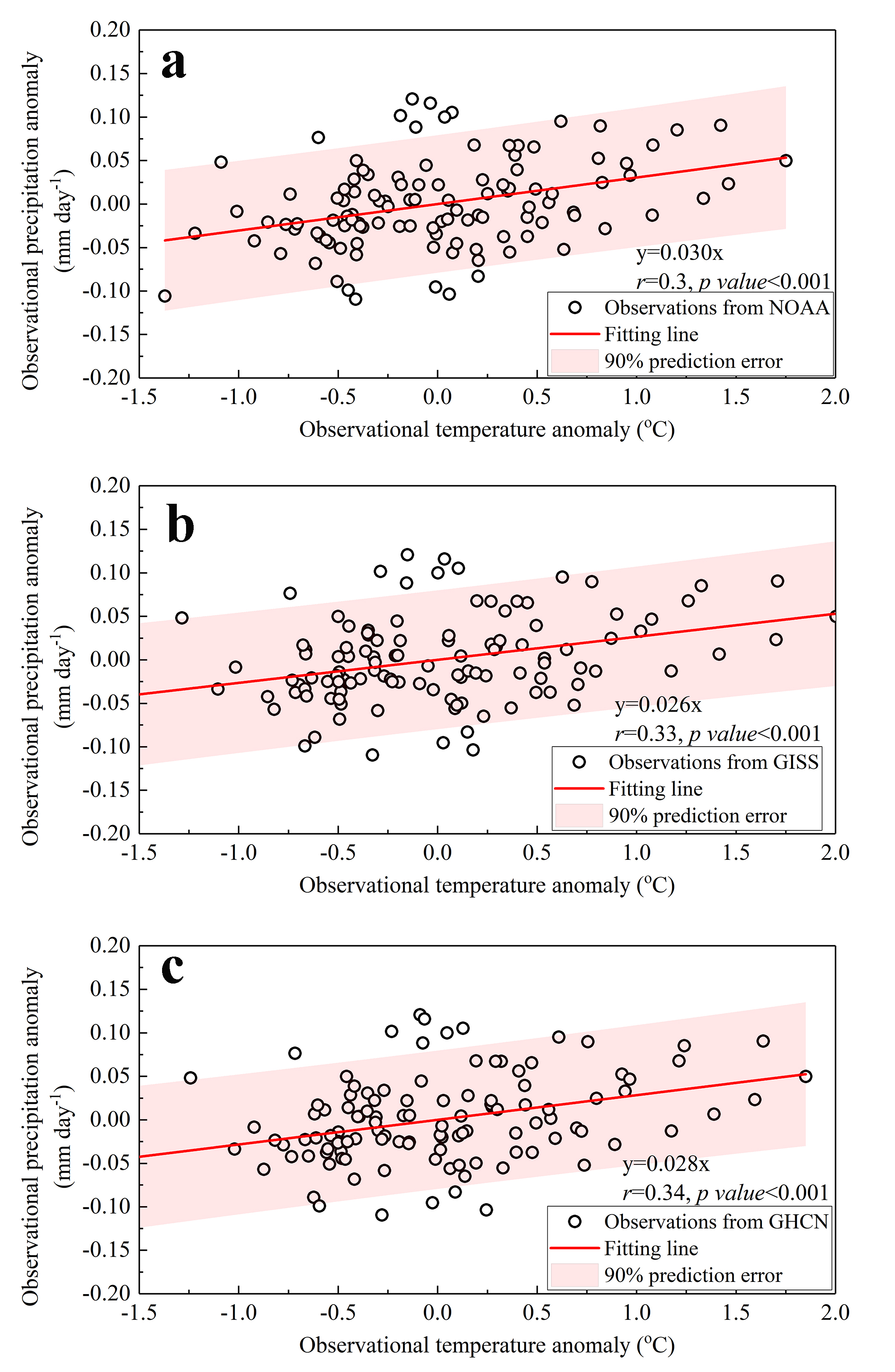

Supplement: S3 Fig — (a), (b) and (c) are relations for the data from NOAA, GHCN, and GISS, respectively. (TIF) [file pone.0301759.s003.tif]

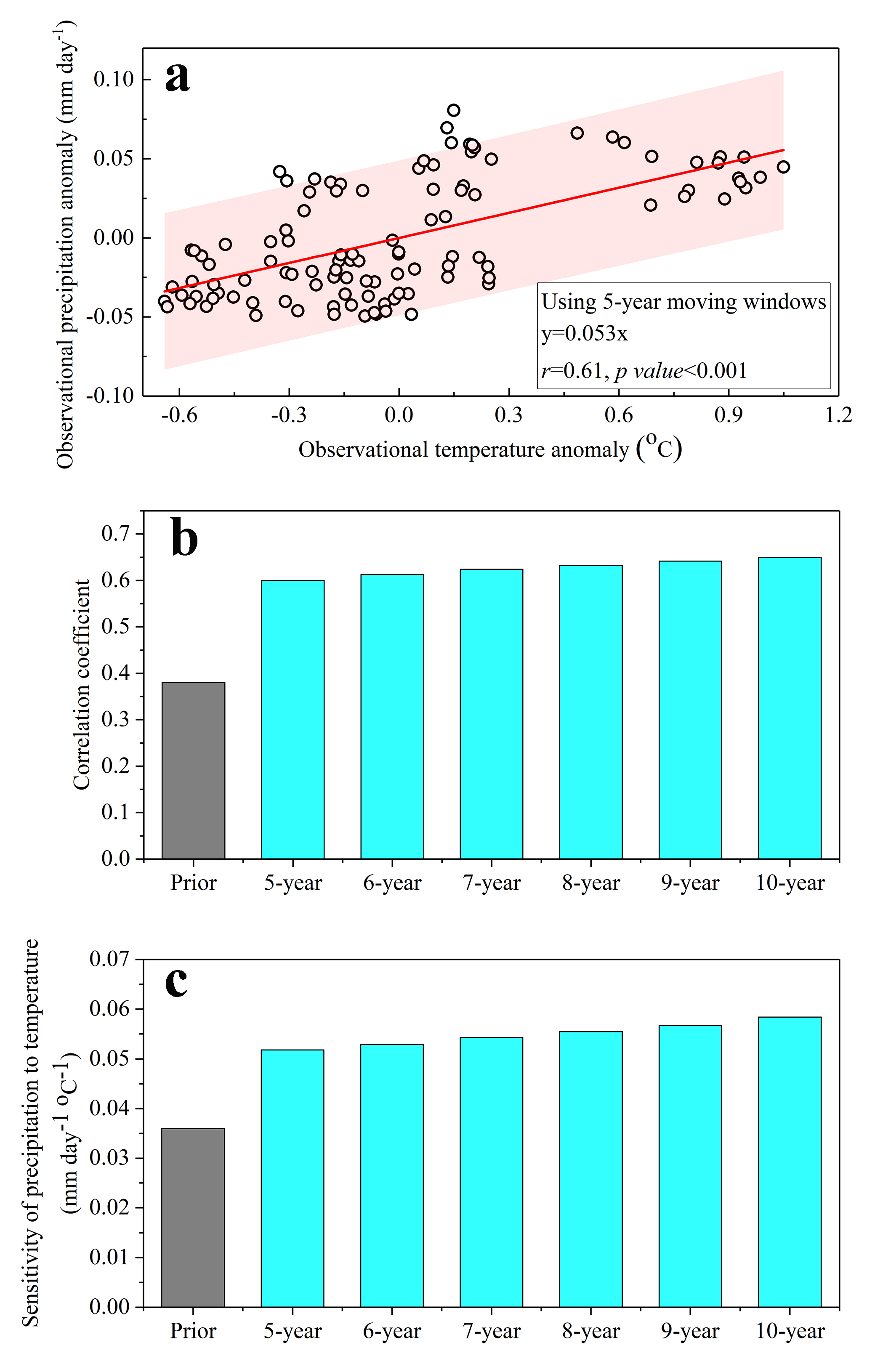

Supplement: S4 Fig — Each circle represents a CMIP6 model. (a), (b) and (c) are the linear relations for the CMIP6 models under SSP126, SSP245, and SSP370, respectively. (TIF) [file pone.0301759.s004.tif]

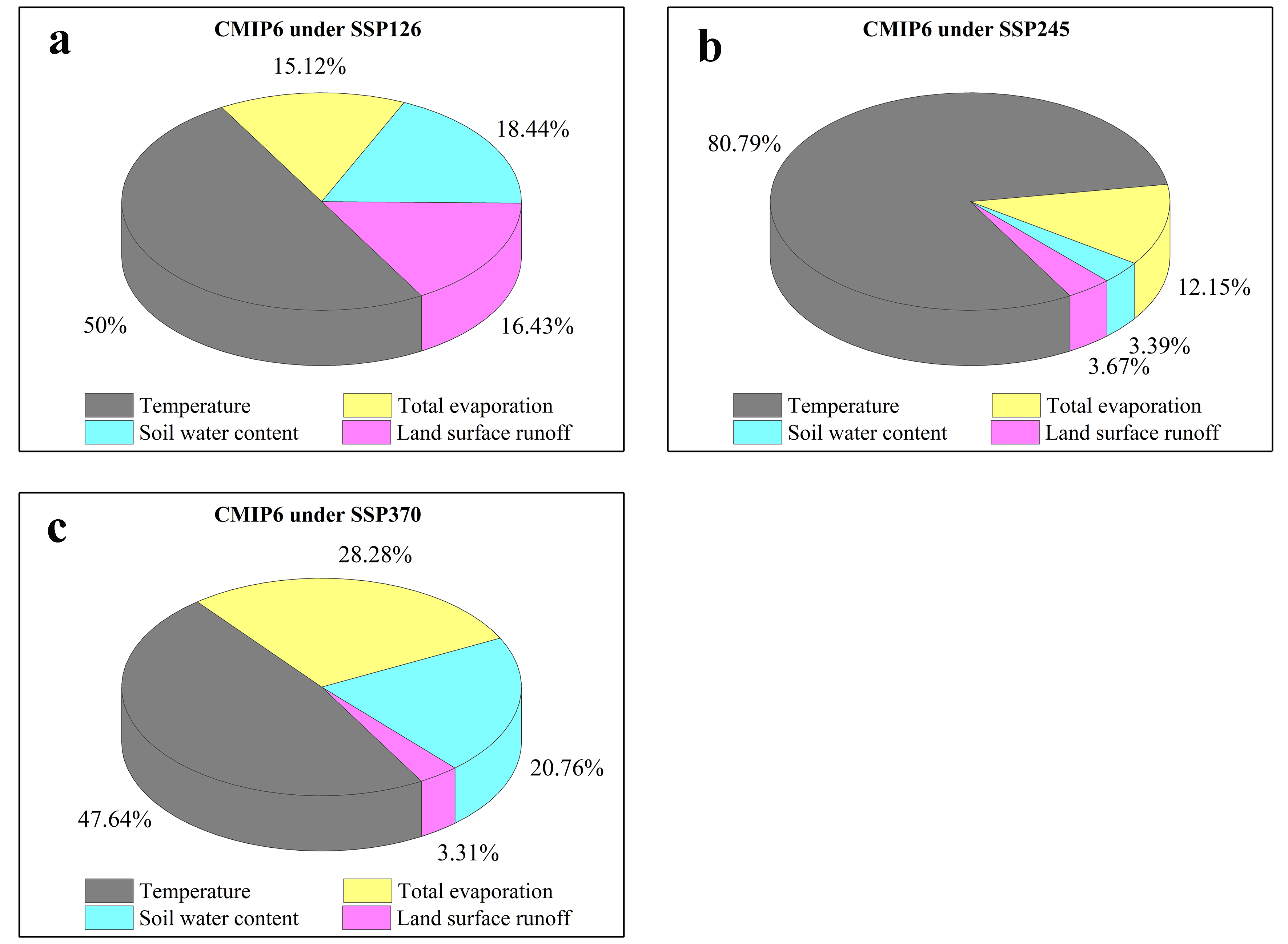

Supplement: S5 Fig — Each circle represents a CMIP5 model. (a) and (b) are the linear relations for the CMIP5 models under RCP45 and RCP85, respectively. (TIF) [file pone.0301759.s005.tif]

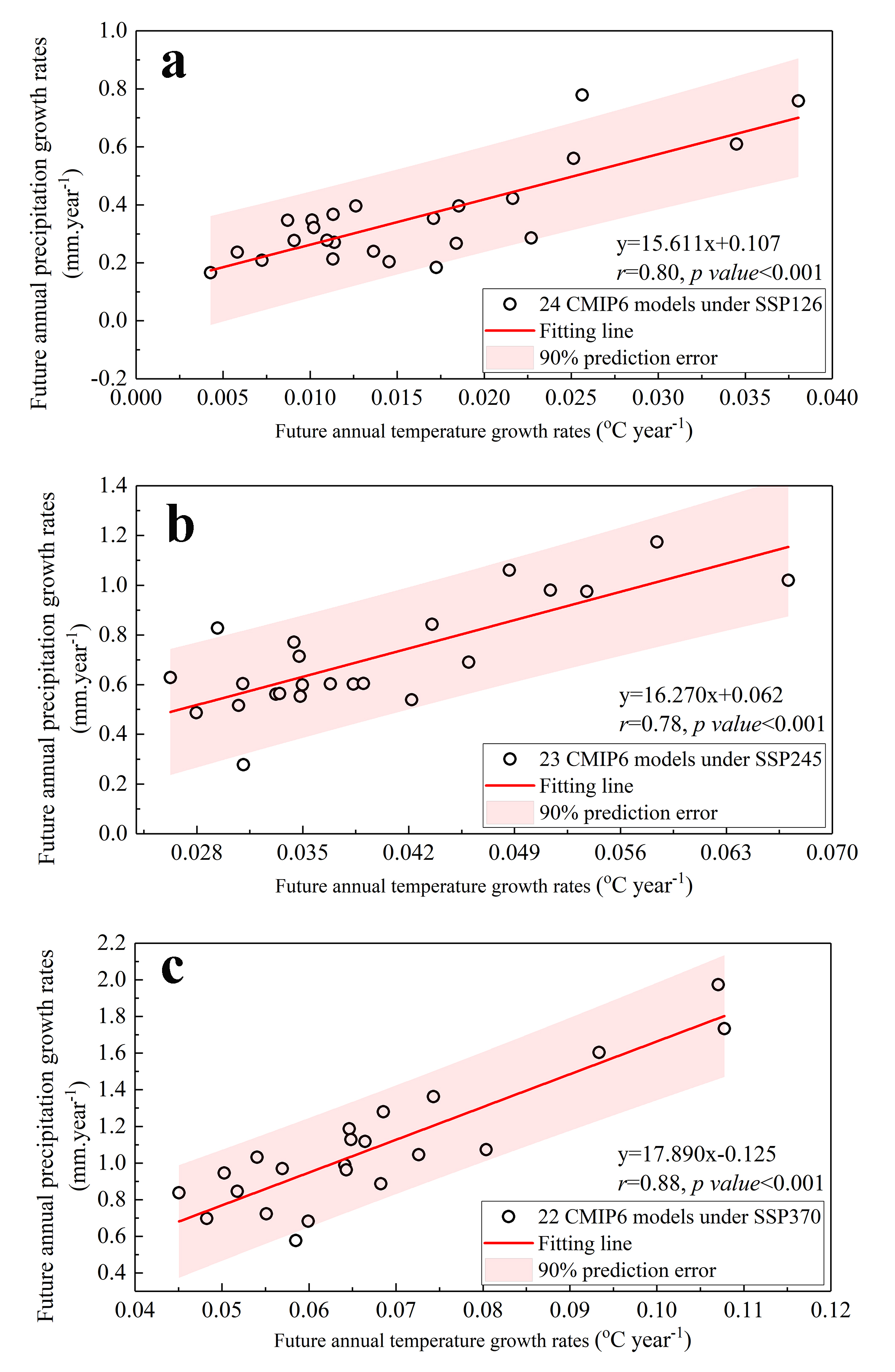

Supplement: S6 Fig — At first, we calculated temperature/precipitation using moving windows with different lengths (5–years) during 1982–2015. Then, the calculated values are used to build the linear relationships. For instance,(a) presents the linear relationship between observed temperature anomaly and observed precipitation anomaly using moving windows with the length of 5 years. Blue histograms in (b) and (c) are the correlation coefficient and the slope values (i.e., sensitivity of precipitation to temperature) respectively, with different lengths of moving windows (5–years). Gray histogram is the values without moving windows (i.e., the values in Fig 1c of the Main Text). (TIF) [file pone.0301759.s006.tif]

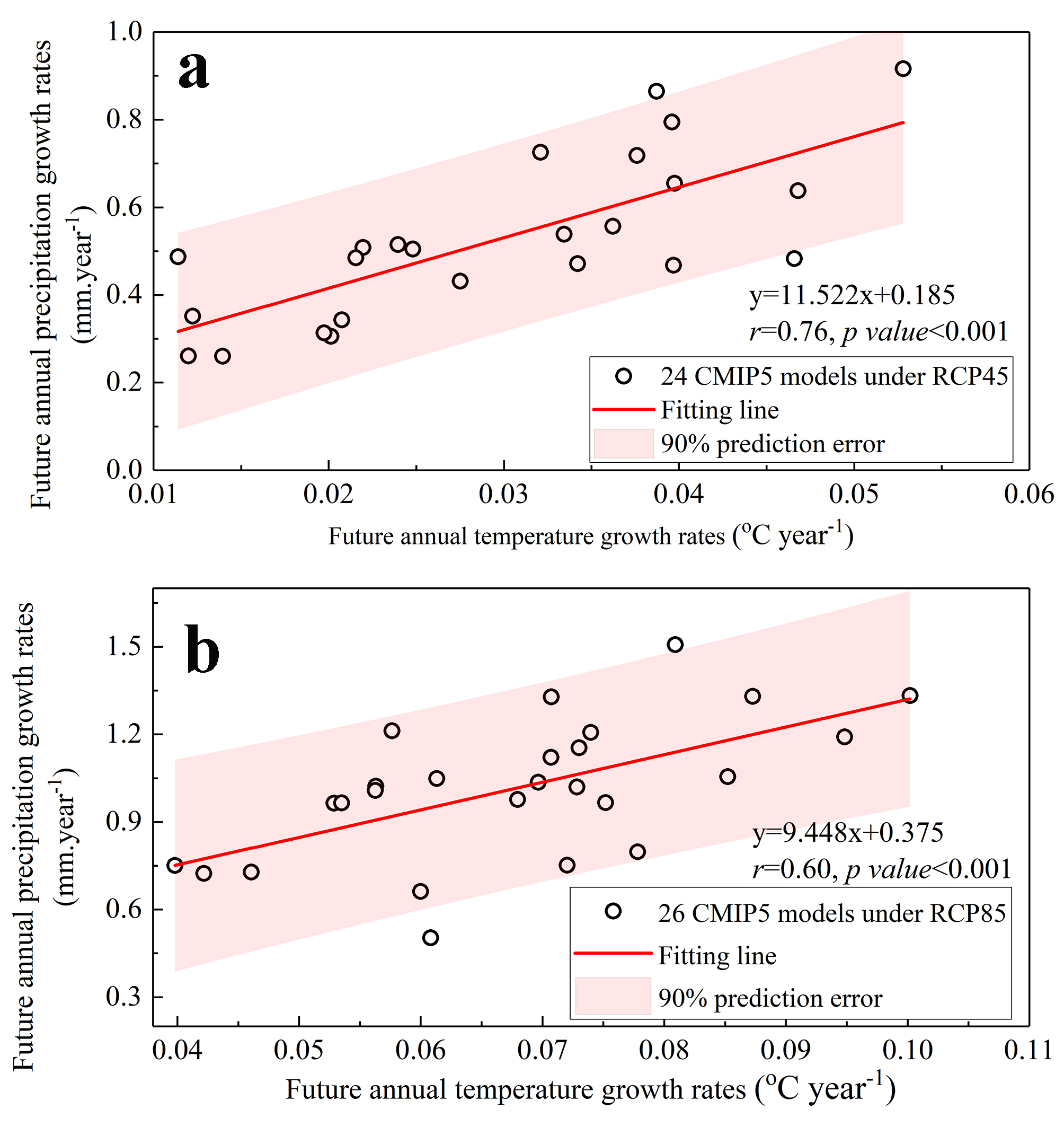

Supplement: S7 Fig — (a), (b) and (c) are the contributions for the emission scenarios of SSP126, SSP245, and SSP370, respectively. (TIF) [file pone.0301759.s007.tif]

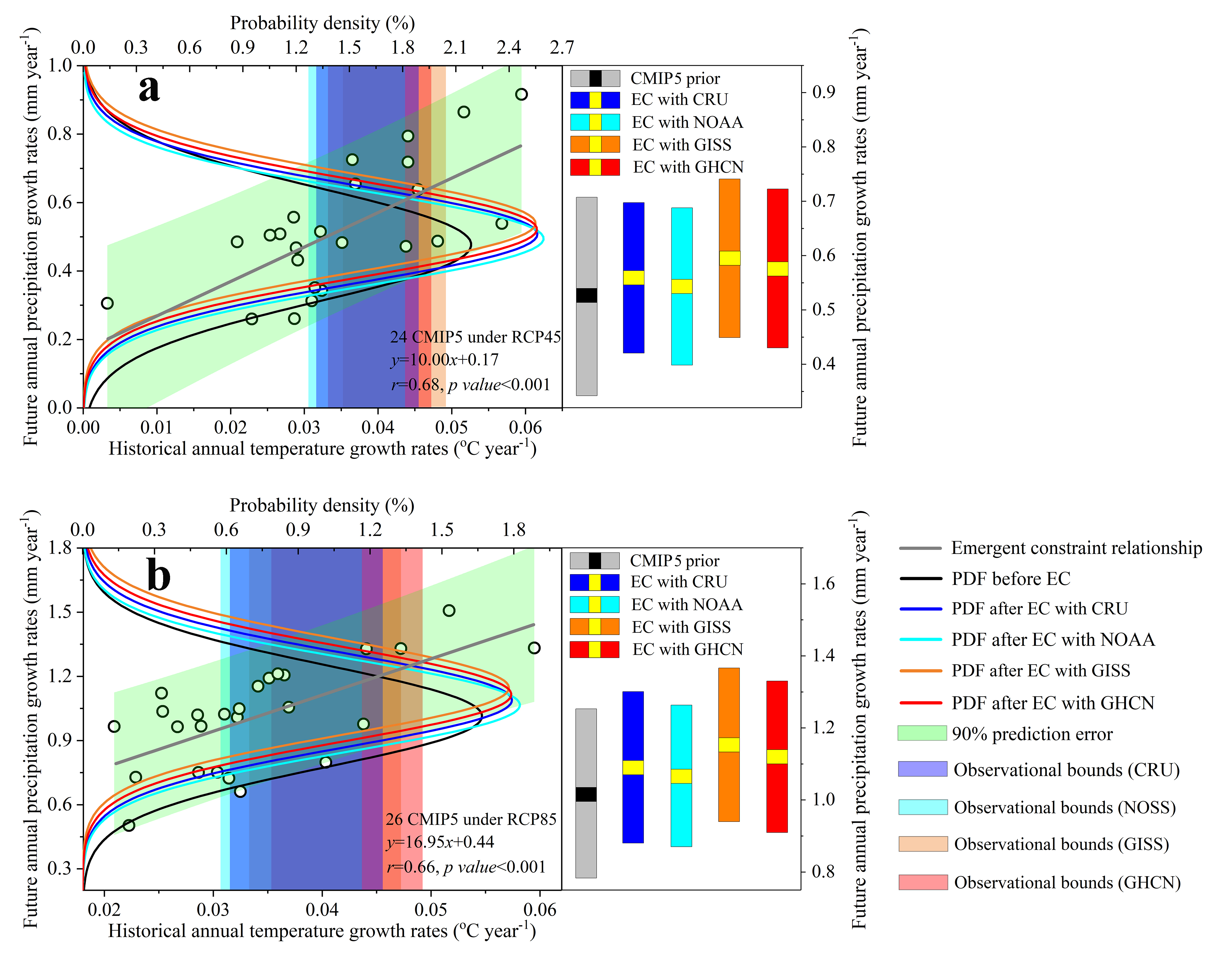

Supplement: S8 Fig — (a) and (b) are the emergent constraint relationships between the simulated historical annual temperature growth rates and the predicted future annual precipitation growth rates across the CMIP5 models under the emission scenarios of RCP45 and RCP85, respectively. Green shading is the 90% prediction error of the linear regression. Each dot represents a model. Four vertical shadings are the observed annual temperature growth rates (Mean ± one standard deviation) from HadCRUT4, NOAA, GISS and GHCN data sets, respectively. Black curves and colorized curves are the PDFs of the future annual precipitation growth rates before and after the emergent constraint, respectively. Gray histograms and colorized histograms in the right panels of S9 Fig the constrained and unconstrained future annual precipitation growth rates (Mean ± one standard deviation), respectively. (TIF) [file pone.0301759.s008.tif]

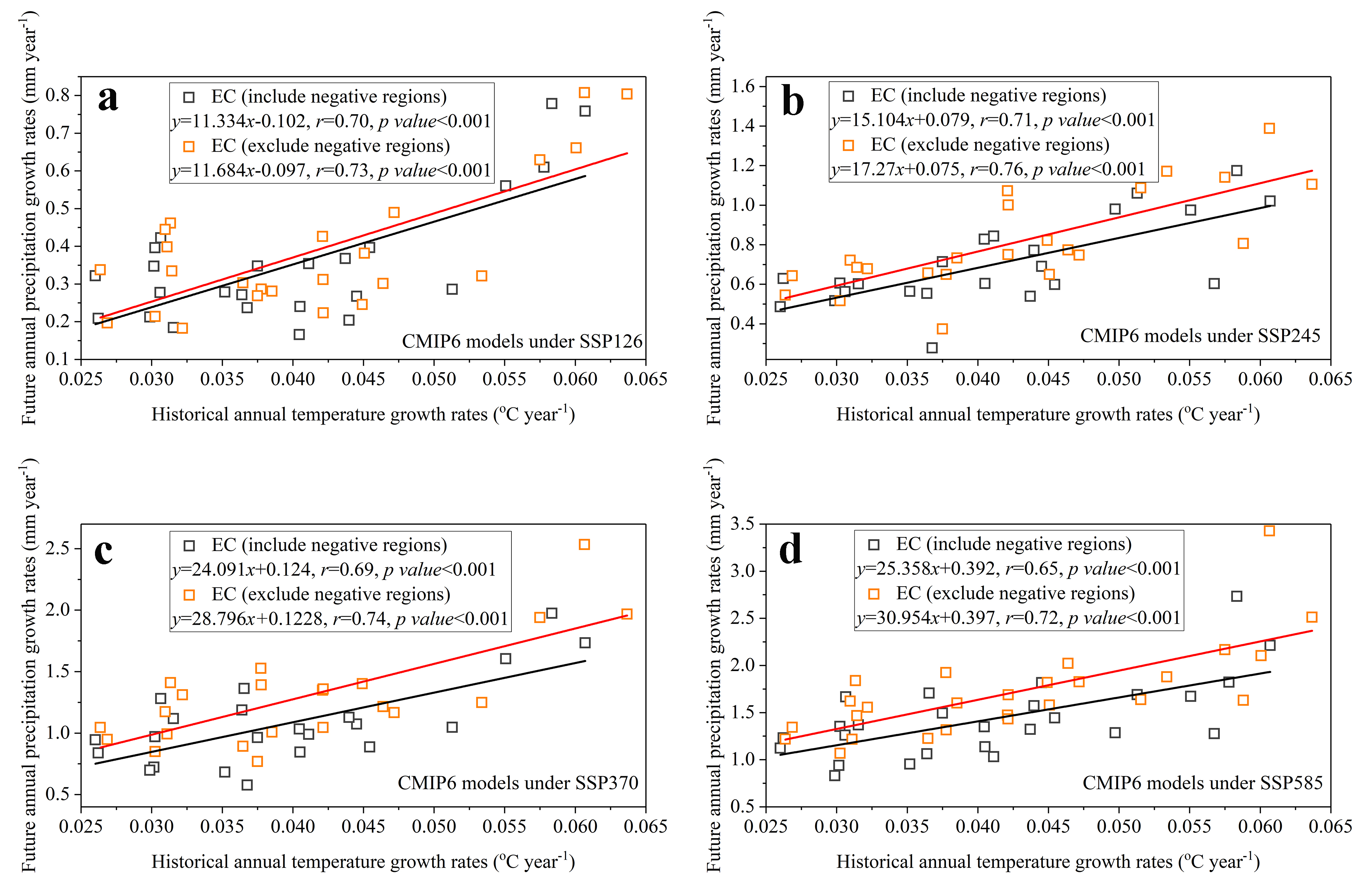

Supplement: S9 Fig — Each dot represents a model. (a), (b), (c) and (d) are the constraint relationships under SSP126, SSP245, SSP370 and SSP585, respectively. Red fitting lines are the emergent constraint relationships after excluding the regions with negative correlations between future precipitation and future temperature (See locations of negative regions in Fig 1d of the Main Text). Dark fitting lines are the emergent constraint relationships including these regions (i.e., the linear relationships in Fig 2 of the Main Text). (TIF) [file pone.0301759.s009.tif]

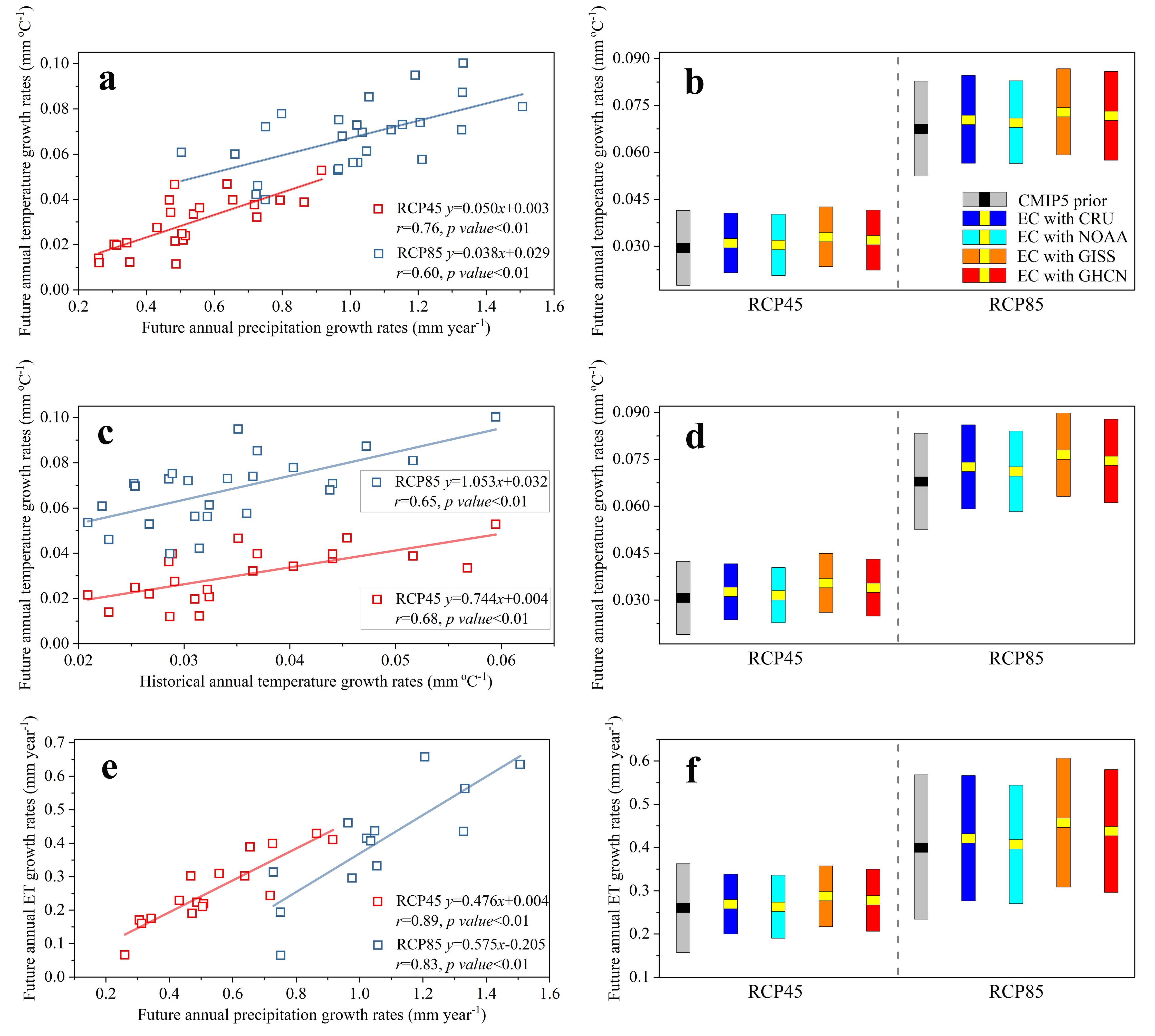

Supplement: S10 Fig — (a) presents the linear relationships between future annual growth rates of precipitation and that of temperature. (b) presents the constrained and the unconstrained future annual temperature growth rates by applying the constrained future annual precipitation growth rates. (c) presents the linear relationships between annual growth rates of historical temperature (1970–2014) and that of future precipitation (2015–2100). (d) presents the constrained and the unconstrained future annual temperature growth rates by applying the observed warming trends. (e) presents the linear relationships between future annual growth rates of precipitation and that of total evaporation. (f) presents the constrained and the unconstrained future annual total evaporation growth rates by applying the constrained future annual precipitation growth rates. (TIF) [file pone.0301759.s010.tif]

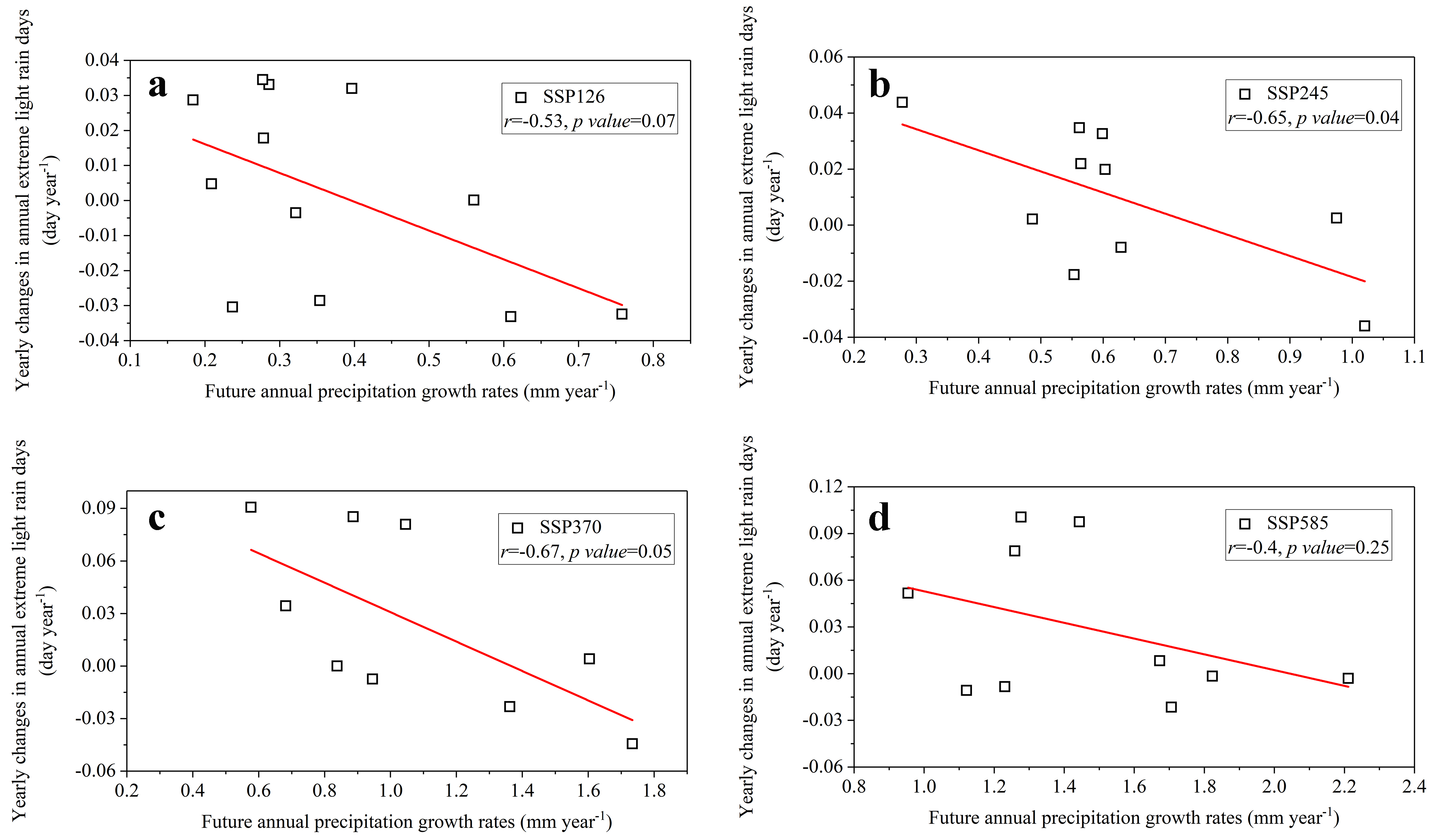

Supplement: S11 Fig — (a), (b), (c) and (d) are the relationships under SSP126, SSP245, SSP370 and SSP585, respectively. Each Circle represents a CMIP6 model. (TIF) [file pone.0301759.s011.tif]
