# Supplementary material for: Uncertainty reduction for precipitation prediction in North America
Source: PLoS One. 2024 May 22;19(5):e0301759. doi: 10.1371/journal.pone.0301759 (PMC11111050; doi:10.1371/journal.pone.0301759)
Supplement: S1 Table — (DOCX) [file pone.0301759.s012.docx]

**S1 Table. Full name of the CMIP6 models for collecting the monthly data of land surface temperature and precipitation during 1970-2100.**

|  | History | SSP126 | SSP245 | SSP370 | SSP585 |
| --- | --- | --- | --- | --- | --- |
| 1 | ACCESS-ESM1-5 | ACCESS-ESM1-5 | ACCESS-ESM1-5 | ACCESS-CM2 | ACCESS-CM2 |
| 2 | ACCESS-CM2 | BCC-CSM2-MR | BCC-CSM2-MR | ACCESS-ESM1-5 | ACCESS-ESM1-5 |
| 3 | BCC-CSM2-MR | CanESM5-CanOE | CanESM5-CanOE | BCC-CSM2-MR | BCC-CSM2-MR |
| 4 | CanESM5-CanOE | CESM2 | CESM2 | CanESM5-CanOE | CanESM5-CanOE |
| 5 | CESM2 | CNRM-CM6-1 | CESM2-WACCM | CESM2 | CESM2 |
| 6 | CESM2-WACCM | CNRM-CM6-1-HR | CNRM-CM6-1 | CNRM-CM6-1 | CESM2-WACCM |
| 7 | CNRM-CM6-1 | CNRM-ESM2-1 | CNRM-ESM2-1 | CNRM-CM6-1-HR | CNRM-CM6-1 |
| 8 | CNRM-CM6-1-HR | FGOALS-f3-L | FGOALS-f3-L | CNRM-ESM2-1 | CNRM-CM6-1-HR |
| 9 | CNRM-ESM2-1 | FIO-ESM-2-0 | FIO-ESM-2-0 | FGOALS-f3-L | CNRM-ESM2-1 |
| 10 | FGOALS-f3-L | GFDL-ESM4 | GFDL-CM4 | GFDL-ESM4 | FGOALS-f3-L |
| 11 | FIO-ESM-2-0 | GISS-E2-1-G | GISS-E2-1-G | GISS-E2-1-G | FIO-ESM-2-0 |
| 12 | GFDL-CM4 | HadGEM3-GC31-LL | INM-CM4-8 | INM-CM4-8 | GFDL-CM4 |
| 13 | GFDL-ESM4 | INM-CM4-8 | INM-CM5-0 | INM-CM5-0 | GFDL-ESM4 |
| 14 | GISS-E2-1-G | INM-CM5-0 | IPSL-CM6A-LR | IPSL-CM6A-LR | GISS-E2-1-G |
| 15 | HadGEM3-GC31-LL | IPSL-CM6A-LR | KACE-1-0-G | KACE-1-0-G | HadGEM3-GC31-LL |
| 16 | INM-CM4-8 | KACE-1-0-G | MCM-UA-1-0 | MCM-UA-1-0 | INM-CM4-8 |
| 17 | INM-CM5-0 | MCM-UA-1-0 | MIROC6 | MIROC6 | INM-CM5-0 |
| 18 | IPSL-CM6A-LR | MIROC6 | MIROC-ES2L | MIROC-ES2L | IPSL-CM6A-LR |
| 19 | KACE-1-0-G | MIROC-ES2L | MPI-ESM1-2-LR | MPI-ESM1-2-LR | KACE-1-0-G |
| 20 | MCM-UA-1-0 | MPI-ESM1-2-LR | MRI-ESM2-0 | MRI-ESM2-0 | MCM-UA-1-0 |
| 21 | MIROC6 | MRI-ESM2-0 | NorESM2-LM | NorESM2-MM | MIROC6 |
| 22 | MIROC-ES2L | NorESM2-LM | NorESM2-MM | UKESM1-0-LL | MIROC-ES2L |
| 23 | MPI-ESM1-2-LR | NorESM2-MM | UKESM1-0-LL |  | MRI-ESM2-0 |
| 24 | MRI-ESM2-0 | UKESM1-0-LL |  |  | NorESM2-LM |
| 25 | NorESM2-LM |  |  |  | NorESM2-MM |
| 26 | NorESM2-MM |  |  |  | UKESM1-0-LL |
| 27 | UKESM1-0-LL |  |  |  |  |
