# Supplementary material for: Uncertainty reduction for precipitation prediction in North America
Source: PLoS One. 2024 May 22;19(5):e0301759. doi: 10.1371/journal.pone.0301759 (PMC11111050; doi:10.1371/journal.pone.0301759)
Supplement: S2 Table — (DOCX) [file pone.0301759.s013.docx]

**S2 Table. Full name of the CMIP5 models for collecting the monthly data of land surface temperature and precipitation during 1970-2100.**

|  | History | RCP45 | RCP85 |
| --- | --- | --- | --- |
| 1 | ACCESS1-0 | ACCESS1-0 | ACCESS1-0 |
| 2 | ACCESS1-0 | CanESM2 | bcc-csm1-1-m |
| 3 | bcc-csm1-1-m | CMCC-CM | BNU-ESM |
| 4 | BNU-ESM | CMCC-CMS | CanESM2 |
| 5 | CanESM2 | CSIRO-Mk3-6-0 | CESM1-WACCM |
| 6 | CESM1-WACCM | CSIRO-Mk3L-1-2 | CMCC-CM |
| 7 | CMCC-CM | FGOALS-g2 | CSIRO-Mk3-6-0 |
| 8 | CMCC-CMS | FGOALS-s2 | EC-EARTH |
| 9 | CSIRO-Mk3-6-0 | GFDL-ESM2G | ensemble |
| 10 | CSIRO-Mk3L-1-2 | GFDL-ESM2M | FGOALS-g2 |
| 11 | EC-EARTH | GISS-E2-H | FIO-ESM |
| 12 | ensemble | GISS-E2-H-CC | GFDL-ESM2G |
| 13 | FGOALS-g2 | GISS-E2-R | GFDL-ESM2M |
| 14 | FGOALS-s2 | GISS-E2-R-CC | GISS-E2-H |
| 15 | FIO-ESM | HadGEM2-AO | GISS-E2-H-CC |
| 16 | GFDL-ESM2G | HadGEM2-CC | GISS-E2-R |
| 17 | GFDL-ESM2M | HadGEM2-ES | GISS-E2-R-CC |
| 18 | GISS-E2-H | inmcm4 | HadGEM2-AO |
| 19 | GISS-E2-H-CC | IPSL-CM5B-LR | HadGEM2-ES |
| 20 | GISS-E2-R | MIROC5 | inmcm4 |
| 21 | GISS-E2-R-CC | MPI-ESM-LR | IPSL-CM5A-LR |
| 22 | HadGEM2-AO | MPI-ESM-MR | IPSL-CM5A-MR |
| 23 | HadGEM2-CC | NorESM1-M | IPSL-CM5B-LR |
| 24 | HadGEM2-ES | NorESM1-ME | MPI-ESM-LR |
| 25 | inmcm4 |  | NorESM1-M |
| 26 | IPSL-CM5A-LR |  | NorESM1-ME |
| 27 | IPSL-CM5A-MR |  |  |
| 28 | IPSL-CM5B-LR |  |  |
| 29 | MIROC5 |  |  |
| 30 | MPI-ESM-LR |  |  |
| 31 | MPI-ESM-MR |  |  |
| 32 | NorESM1-M |  |  |
| 33 | NorESM1-ME |  |  |
