# Supplementary material for: Uncertainty reduction for precipitation prediction in North America
Source: PLoS One. 2024 May 22;19(5):e0301759. doi: 10.1371/journal.pone.0301759 (PMC11111050; doi:10.1371/journal.pone.0301759)
Supplement: S3 Table — (DOCX) [file pone.0301759.s014.docx]

**S3 Table. Full name of the CMIP6 models for collecting the monthly data of total evaporation under 2015-2100.**

|  | SSP126 | SSP245 | SSP370 | SSP585 |
| --- | --- | --- | --- | --- |
| 1 | ACCESS-ESM1-5 | BCC-CSM2-MR | BCC-CSM2-MR | ACCESS-ESM1-5 |
| 2 | BCC-CSM2-MR | CanESM5-CanOE | CanESM5-CanOE | BCC-CSM2-MR |
| 3 | CanESM5-CanOE | CESM2 | CESM2 | CanESM5-CanOE |
| 4 | CNRM-CM6-1 | CNRM-CM6-1 | CNRM-CM6-1 | CESM2-WACCM |
| 5 | CNRM-CM6-1-HR | CNRM-ESM2-1 | CNRM-CM6-1-HR | CNRM-CM6-1 |
| 6 | CNRM-ESM2-1 | GISS-E2-1-G | CNRM-ESM2-1 | CNRM-CM6-1-HR |
| 7 | GISS-E2-1-G | INM-CM4-8 | GISS-E2-1-G | CNRM-ESM2-1 |
| 8 | INM-CM4-8 | INM-CM5-0 | INM-CM4-8 | GISS-E2-1-G |
| 9 | INM-CM5-0 | IPSL-CM6A-LR | INM-CM5-0 | INM-CM4-8 |
| 10 | IPSL-CM6A-LR | MCM-UA-1-0 | IPSL-CM6A-LR | INM-CM5-0 |
| 11 | KACE-1-0-G | MIROC6 | KACE-1-0-G | IPSL-CM6A-LR |
| 12 | MCM-UA-1-0 | MIROC-ES2L | MCM-UA-1-0 | KACE-1-0-G |
| 13 | MIROC6 | MPI-ESM1-2-LR | MIROC6 | MCM-UA-1-0 |
| 14 | MIROC-ES2L | MRI-ESM2-0 | MIROC-ES2L | MIROC6 |
| 15 | MRI-ESM2-0 | NorESM2-MM | MPI-ESM1-2-LR | MIROC-ES2L |
| 16 |  |  | MRI-ESM2-0 | MRI-ESM2-0 |
| 17 |  |  | NorESM2-MM | NorESM2-MM |
| 18 |  |  |  |  |
| 19 |  |  |  |  |
