# Supplementary material for: Uncertainty reduction for precipitation prediction in North America
Source: PLoS One. 2024 May 22;19(5):e0301759. doi: 10.1371/journal.pone.0301759 (PMC11111050; doi:10.1371/journal.pone.0301759)
Supplement: S4 Table — (DOCX) [file pone.0301759.s015.docx]

**S4 Table. Full name of the CMIP6 models for collecting the monthly data of soil water content during 2015-2100.**

|  | SSP126 | SSP245 | SSP370 | SSP585 |
| --- | --- | --- | --- | --- |
| 1 | BCC-CSM2-MR | ACCESS-CM2 | ACCESS-CM2 | ACCESS-CM2 |
| 2 | CanESM5 | ACCESS-ESM1-5 | ACCESS-ESM1-5 | ACCESS-ESM1-5 |
| 3 | CanESM5-CanOE | BCC-CSM2-MR | BCC-CSM2-MR | BCC-CSM2-MR |
| 4 | CNRM-CM6-1-HR | CanESM5 | CanESM5 | CanESM5 |
| 5 | FGOALS-f3-L | CanESM5-CanOE | CanESM5-CanOE | CanESM5-CanOE |
| 6 | HadGEM3-GC31-LL | CNRM-CM6-1 | CNRM-CM6-1 | CNRM-CM6-1 |
| 7 | INM-CM4-8 | CNRM-CM6-1-HR | CNRM-CM6-1-HR | CNRM-CM6-1-HR |
| 8 | INM-CM5-0 | CNRM-ESM2-1 | CNRM-ESM2-1 | CNRM-ESM2-1 |
| 9 | IPSL-CM6A-LR | FGOALS-f3-L | FGOALS-f3-L | FGOALS-f3-L |
| 10 | MIROC6 | HadGEM3-GC31-LL | INM-CM4-8 | HadGEM3-GC31-LL |
| 11 | MIROC-ES2L | INM-CM4-8 | INM-CM5-0 | INM-CM4-8 |
| 12 | MPI-ESM1-2-LR | INM-CM5-0 | IPSL-CM6A-LR | INM-CM5-0 |
| 13 | MRI-ESM2-0 | IPSL-CM6A-LR | MIROC6 | IPSL-CM6A-LR |
| 14 | NorESM2-LM | MIROC6 | MIROC-ES2L | MIROC6 |
| 15 | NorESM2-MM | MIROC-ES2L | MPI-ESM1-2-LR | MIROC-ES2L |
| 16 | UKESM1-0-LL | MPI-ESM1-2-LR | MRI-ESM2-0 | MPI-ESM1-2-LR |
| 17 |  | MRI-ESM2-0 | NorESM2-LM | MRI-ESM2-0 |
| 18 |  | NorESM2-LM | NorESM2-MM | NorESM2-LM |
| 19 |  | NorESM2-MM | UKESM1-0-LL | NorESM2-MM |
| 20 |  | UKESM1-0-LL |  | UKESM1-0-LL |
