# Supplementary material for: Uncertainty reduction for precipitation prediction in North America
Source: PLoS One. 2024 May 22;19(5):e0301759. doi: 10.1371/journal.pone.0301759 (PMC11111050; doi:10.1371/journal.pone.0301759)
Supplement: S5 Table — (DOCX) [file pone.0301759.s016.docx]

**S5 Table. Full name of the CMIP6 models for collecting the monthly data of land surface runoff during 1970-2100.**

|  | SSP126 | SSP245 | SSP370 | SSP585 |
| --- | --- | --- | --- | --- |
| 1 | ACCESS-CM2 | ACCESS-CM2 | ACCESS-CM2 | ACCESS-ESM1-5 |
| 2 | ACCESS-ESM1-5 | ACCESS-ESM1-5 | ACCESS-ESM1-5 | BCC-CSM2-MR |
| 3 | BCC-CSM2-MR | BCC-CSM2-MR | BCC-CSM2-MR | CanESM5-CanOE |
| 4 | CESM2 | CanESM5 | CESM2-WACCM | CESM2 |
| 5 | CESM2-WACCM | CanESM5-CanOE | CESM2 | CESM2-WACCM |
| 6 | CNRM-CM6-1 | CESM2 | CNRM-CM6-1-HR | CNRM-CM6-1-HR |
| 7 | CNRM-CM6-1-HR | CESM2-WACCM | CNRM-CM6-1 | CNRM-ESM2-1 |
| 8 | CNRM-ESM2-1 | CNRM-CM6-1 | CNRM-ESM2-1 | FIO-ESM-2-0 |
| 9 | FIO-ESM-2-0 | CNRM-CM6-1-HR | CanESM5-CanOE | GISS-E2-1-G |
| 10 | GISS-E2-1-G | CNRM-ESM2-1 | CanESM5 | INM-CM4-8 |
| 11 | HadGEM3-GC31-LL | FIO-ESM-2-0 | GISS-E2-1-G | INM-CM5-0 |
| 12 | INM-CM4-8 | GISS-E2-1-G | INM-CM4-8 | IPSL-CM6A-LR |
| 13 | INM-CM5-0 | HadGEM3-GC31-LL | INM-CM5-0 | MIROC6 |
| 14 | IPSL-CM6A-LR | INM-CM4-8 | IPSL-CM6A-LR | MIROC-ES2L |
| 15 | MCM-UA-1-0 | INM-CM5-0 | MCM-UA-1-0 | MPI-ESM1-2-LR |
| 16 | MIROC6 | IPSL-CM6A-LR | MIROC-ES2L | NorESM2-LM |
| 17 | MIROC-ES2L | MCM-UA-1-0 | MIROC6 | NorESM2-MM |
| 18 | MRI-ESM2-0 | MIROC6 | MPI-ESM1-2-LR |  |
| 19 | NorESM2-LM | MIROC-ES2L | MRI-ESM2-0 |  |
| 20 | NorESM2-MM | MPI-ESM1-2-LR | NorESM2-LM |  |
| 21 | UKESM1-0-LL | MRI-ESM2-0 | NorESM2-MM |  |
| 22 |  | NorESM2-LM | UKESM1-0-LL |  |
| 23 |  | NorESM2-MM |  |  |
| 24 |  | UKESM1-0-LL |  |  |
