# Supplementary material for: Uncertainty reduction for precipitation prediction in North America
Source: PLoS One. 2024 May 22;19(5):e0301759. doi: 10.1371/journal.pone.0301759 (PMC11111050; doi:10.1371/journal.pone.0301759)
Supplement: S6 Table — Overestimated future precipitation increase = │constrained precipitation–unconstrained precipitation│/unconstrained precipitation; Reduced uncertainty = │constrained standard deviation–unconstrained standard deviation│/unconstrained standard deviation. (DOCX) [file pone.0301759.s017.docx]

**S6 Table**. Emergent constraint on the future annual precipitation growth rates in North America for the period of 2015-2100 based on CMIP6 projections. Overestimated future precipitation increase=│constrained precipitation－unconstrained precipitation│/unconstrained precipitation; Reduced uncertainty=│constrained standard deviation－unconstrained standard deviation│/unconstrained standard deviation;

|  | Observed annual temperature growth rates ± one standard deviation  (℃ year^-1^) |  | Future annual precipitation growth rates  before emergent constraint | | Future annual precipitation growth rates  after emergent constraint | | Overestimated future  precipitation increase  (%) | Reduced uncertainty (%) |
| --- | --- | --- | --- | --- | --- | --- | --- | --- |
|  |  |  | Mean value  (mm year^-1^) | one standard deviation | Mean value  (mm year^-1^) | one standard deviation |  |  |
| HadCRUT4 | 0.0357 ± 0.0050 | SSP126 | 0.3538 | 0.1676 | 0.3028 | 0.1227 | 14.4% | 26.8% |
|  |  | SSP245 | 0.7043 | 0.2182 | 0.6178 | 0.1678 | 12.3% | 23.1% |
|  |  | SSP370 | 1.0752 | 0.3470 | 0.9834 | 0.2834 | 8.5% | 18.3% |
|  |  | SSP585 | 1.4364 | 0.4165 | 1.2970 | 0.3370 | 9.7% | 19.1% |
| NOAA | 0.0346 ± 0.0048 | SSP126 | 0.3538 | 0.1676 | 0.2903 | 0.1303 | 17.9% | 22.3% |
|  |  | SSP245 | 0.7043 | 0.2182 | 0.6012 | 0.1712 | 14.6% | 21.5% |
|  |  | SSP370 | 1.0752 | 0.3470 | 0.9569 | 0.2869 | 11.0% | 17.3% |
|  |  | SSP585 | 1.4364 | 0.4165 | 1.2691 | 0.3591 | 11.6% | 13.8% |
| GISS | 0.0394 ± 0.0056 | SSP126 | 0.3538 | 0.1676 | 0.3455 | 0.1155 | 2.3% | 31.1% |
|  |  | SSP245 | 0.7043 | 0.2182 | 0.6748 | 0.1548 | 4.2% | 29.1% |
|  |  | SSP370 | 1.0752 | 0.3470 | 1.0742 | 0.2742 | 0.1% | 21.0% |
|  |  | SSP585 | 1.4364 | 0.4165 | 1.3926 | 0.3326 | 3.0% | 20.1% |
| GHCN | 0.0365 ± 0.0050 | SSP126 | 0.3538 | 0.1676 | 0.3126 | 0.1226 | 11.6% | 26.8% |
|  |  | SSP245 | 0.7043 | 0.2182 | 0.6310 | 0.1610 | 10.4% | 26.2% |
|  |  | SSP370 | 1.0752 | 0.3470 | 1.0044 | 0.2644 | 6.6% | 23.8% |
|  |  | SSP585 | 1.4364 | 0.4165 | 1.3190 | 0.3390 | 8.2% | 18.6% |
