# Supplementary material for: Uncertainty reduction for precipitation prediction in North America
Source: PLoS One. 2024 May 22;19(5):e0301759. doi: 10.1371/journal.pone.0301759 (PMC11111050; doi:10.1371/journal.pone.0301759)
Supplement: S7 Table — (DOCX) [file pone.0301759.s018.docx]

**S7 Table**. Emergent constraint on the future annual precipitation growth rates in North America for the period of 2006-2100 based on CMIP5 projections .

|  | Observed annual temperature growth rates ± one standard deviation  (℃ year^-1^) |  | Future annual precipitation growth rates  before emergent constraint | | Future annual precipitation growth rates  after emergent constraint | | Underestimated future  precipitation increase  (%) | Reduced uncertainty (%) |
| --- | --- | --- | --- | --- | --- | --- | --- | --- |
|  |  |  | Mean value  (mm year^-1^) | one standard deviation | Mean value  (mm year^-1^) | one standard deviation |  |  |
| HadCRUT4 | 0.0385 ± 0.0070 | RCP45 | 0.5248 | 0.1825 | 0.5587 | 0.1386 | 6.5% | 24.1% |
|  |  | RCP85 | 1.0139 | 0.2393 | 1.0902 | 0.2102 | 7.5% | 12.2% |
| NOAA | 0.0371 ± 0.0065 | RCP45 | 0.5248 | 0.1825 | 0.5445 | 0.1444 | 3.8% | 20.9% |
|  |  | RCP85 | 1.0139 | 0.2393 | 1.0661 | 0.1961 | 5.1% | 18.1% |
| GISS | 0.0422 ± 0.0070 | RCP45 | 0.5248 | 0.1825 | 0.5959 | 0.1458 | 13.5% | 20.1% |
|  |  | RCP85 | 1.0139 | 0.2393 | 1.1533 | 0.2132 | 13.7% | 10.9% |
| GHCN | 0.0403 ± 0.0070 | RCP45 | 0.5248 | 0.1825 | 0.5761 | 0.1460 | 9.8% | 20.0% |
|  |  | RCP85 | 1.0139 | 0.2393 | 1.1197 | 0.2097 | 10.4% | 12.4% |
