# Supplementary material for: Uncertainty reduction for precipitation prediction in North America
Source: PLoS One. 2024 May 22;19(5):e0301759. doi: 10.1371/journal.pone.0301759 (PMC11111050; doi:10.1371/journal.pone.0301759)
Supplement: S8 Table — (DOCX) [file pone.0301759.s019.docx]

**S8 Table**. Constraint on the future annual temperature growth rates in North America for the period of 2015-2100 based on CMIP6 projections by using the constrained future annual precipitation growth rates.

|  | Constrained future annual precipitation growth rates± one standard deviation  (mm year^-1^) | | Future annual temperature growth rates  before emergent constraint | | Future annual temperature growth rates  after emergent constraint | | Overestimated future temperature increase  (%) | Reduced uncertainty (%) |
| --- | --- | --- | --- | --- | --- | --- | --- | --- |
|  |  |  | Mean value  (℃ year^-1^) | one standard deviation | Mean value  (℃ year^-1^) | one standard deviation |  |  |
| HadCRUT4 | SSP126 | 0.3028 ± 0.1227 | 0.0158 | 0.0086 | 0.0137 | 0.0060 | 13.3% | 30.2 % |
|  | SSP245 | 0.6178 ± 0.1678 | 0.0395 | 0.0105 | 0.0362 | 0.0082 | 8.4 % | 21.9 % |
|  | SSP370 | 0.9834 ± 0.2834 | 0.0671 | 0.0171 | 0.0631 | 0.0138 | 6.0 % | 19.3 % |
|  | SSP585 | 1.2970 ± 0.3370 | 0.0897 | 0.0210 | 0.0841 | 0.0179 | 6.2 % | 14.8 % |
| NOAA | SSP126 | 0.2903 ± 0.1303 | 0.0158 | 0.0086 | 0.0132 | 0.0065 | 16.5 % | 24.4 % |
|  | SSP245 | 0.6012 ± 0.1712 | 0.0395 | 0.0105 | 0.0356 | 0.0080 | 9.9 % | 23.8 % |
|  | SSP370 | 0.9569 ± 0.2869 | 0.0671 | 0.0171 | 0.0619 | 0.0138 | 7.7 % | 19.3 % |
|  | SSP585 | 1.2691 ± 0.3591 | 0.0897 | 0.0210 | 0.0830 | 0.0185 | 7.5 % | 11.9 % |
| GISS | SSP126 | 0.3455 ± 0.1155 | 0.0158 | 0.0086 | 0.0155 | 0.0057 | 1.9 % | 33.7 % |
|  | SSP245 | 0.6748 ± 0.1548 | 0.0395 | 0.0105 | 0.0383 | 0.0079 | 3.0 % | 24.8 % |
|  | SSP370 | 1.0742 ± 0.2742 | 0.0671 | 0.0171 | 0.0670 | 0.0134 | 0.1 % | 21.6 % |
|  | SSP585 | 1.3926 ± 0.3326 | 0.0897 | 0.0210 | 0.0879 | 0.0175 | 2.0 % | 16.7 % |
| GHCN | SSP126 | 0.3126 ± 0.1226 | 0.0158 | 0.0086 | 0.0142 | 0.0062 | 10.1 % | 27.9 % |
|  | SSP245 | 0.6310 ± 0.1610 | 0.0395 | 0.0105 | 0.0367 | 0.0078 | 7.1 % | 25.7 % |
|  | SSP370 | 1.0044 ± 0.2644 | 0.0671 | 0.0171 | 0.0640 | 0.0130 | 4.6 % | 24.0 % |
|  | SSP585 | 1.3190 ± 0.3390 | 0.0897 | 0.0210 | 0.0849 | 0.0178 | 5.4 % | 15.2 % |
