# Supplementary material for: Uncertainty reduction for precipitation prediction in North America
Source: PLoS One. 2024 May 22;19(5):e0301759. doi: 10.1371/journal.pone.0301759 (PMC11111050; doi:10.1371/journal.pone.0301759)
Supplement: S9 Table — (DOCX) [file pone.0301759.s020.docx]

**S9 Table**. Constraint on the future annual temperature in North America for the period of 2015-2100 based on CMIP5 projections by using constrained future annual precipitation growth rates.

|  | Constrained future annual precipitation growth rates± one standard deviation  (mm year^-1^) | | Future annual temperature growth rates  before emergent constraint | | Future annual temperature growth rates  after emergent constraint | | Overestimated future temperature increase  (%) | Reduced uncertainty (%) |
| --- | --- | --- | --- | --- | --- | --- | --- | --- |
|  |  |  | Mean value  (℃ year^-1^) | one standard deviation | Mean value  (℃ year^-1^) | one standard deviation |  |  |
| HadCRUT4 | RCP45 | 0.5587 ± 0.1386 | 0.0295 | 0.0120 | 0.0312 | 0.0095 | 5.8 % | 20.8 % |
|  | RCP85 | 1.0902 ± 0.2102 | 0.0677 | 0.0152 | 0.0706 | 0.0140 | 4.3 % | 7.9 % |
| NOAA | RCP45 | 0.5445 ± 0.1444 | 0.0295 | 0.0120 | 0.0305 | 0.0099 | 3.4 % | 17.5 % |
|  | RCP85 | 1.0661 ± 0.1961 | 0.0677 | 0.0152 | 0.0696 | 0.0133 | 2.8 % | 12.5 % |
| GISS | RCP45 | 0.5959 ± 0.1458 | 0.0295 | 0.0120 | 0.0330 | 0.0096 | 11.9 % | 20.0 % |
|  | RCP85 | 1.1533 ± 0.2132 | 0.0677 | 0.0152 | 0.0730 | 0.0137 | 7.8 % | 9.9 % |
| GHCN | RCP45 | 0.5761 ± 0.1460 | 0.0295 | 0.0120 | 0.0320 | 0.0096 | 8.5 % | 20.0 % |
|  | RCP85 | 1.1197 ± 0.2097 | 0.0677 | 0.0152 | 0.0717 | 0.0141 | 5.9 % | 7.2 % |
