# Supplementary material for: Uncertainty reduction for precipitation prediction in North America
Source: PLoS One. 2024 May 22;19(5):e0301759. doi: 10.1371/journal.pone.0301759 (PMC11111050; doi:10.1371/journal.pone.0301759)
Supplement: S10 Table — (DOCX) [file pone.0301759.s021.docx]

**S10 Table**. Constraint on the future annual temperature growth rates in North America for the period of 2015-2100 based on CMIP6 projections by using the observed annual temperature growth rates.

|  | Observed annual temperature growth rates ± one standard deviation  (℃ year^-1^) |  | Future annual temperature growth rates  before emergent constraint | | Future annual temperature growth rates  after emergent constraint | | Overestimated future  temperature increase  (%) | Reduced uncertainty (%) |
| --- | --- | --- | --- | --- | --- | --- | --- | --- |
|  |  |  | Mean value  (℃ year^-1^) | one standard deviation | Mean value  (℃ year^-1^) | one standard deviation |  |  |
| HadCRUT4 | 0.0357 ± 0.0050 | SSP126 | 0.0156 | 0.0087 | 0.0122 | 0.0049 | 21.8% | 43.7% |
|  |  | SSP245 | 0.0396 | 0.0107 | 0.0349 | 0.0061 | 11.9% | 43.0% |
|  |  | SSP370 | 0.0671 | 0.0171 | 0.0615 | 0.0104 | 8.3% | 39.2% |
|  |  | SSP585 | 0.0889 | 0.0209 | 0.0801 | 0.0137 | 9.9% | 34.4% |
| NOAA | 0.0346 ± 0.0048 | SSP126 | 0.0156 | 0.0087 | 0.0114 | 0.0050 | 26.9% | 42.5% |
|  |  | SSP245 | 0.0396 | 0.0107 | 0.0339 | 0.0060 | 14.4% | 43.9% |
|  |  | SSP370 | 0.0671 | 0.0171 | 0.0599 | 0.0104 | 10.7% | 39.2% |
|  |  | SSP585 | 0.0889 | 0.0209 | 0.0783 | 0.0137 | 11.9% | 34.4% |
| GISS | 0.0394 ± 0.0056 | SSP126 | 0.0156 | 0.0087 | 0.0148 | 0.0049 | 5.1% | 43.7% |
|  |  | SSP245 | 0.0396 | 0.0107 | 0.0384 | 0.0067 | 3.0% | 37.4% |
|  |  | SSP370 | 0.0671 | 0.0171 | 0.0670 | 0.0111 | 0.1% | 35.1% |
|  |  | SSP585 | 0.0889 | 0.0209 | 0.0863 | 0.0143 | 2.9% | 31.6% |
| GHCN | 0.0365 ± 0.0050 | SSP126 | 0.0156 | 0.0087 | 0.0128 | 0.0047 | 17.9% | 46.0% |
|  |  | SSP245 | 0.0396 | 0.0107 | 0.0357 | 0.0060 | 9.8% | 43.9% |
|  |  | SSP370 | 0.0671 | 0.0171 | 0.0628 | 0.0105 | 6.4% | 38.6% |
|  |  | SSP585 | 0.0889 | 0.0209 | 0.0816 | 0.0134 | 8.2% | 35.9% |
