# Supplementary material for: Uncertainty reduction for precipitation prediction in North America
Source: PLoS One. 2024 May 22;19(5):e0301759. doi: 10.1371/journal.pone.0301759 (PMC11111050; doi:10.1371/journal.pone.0301759)
Supplement: S11 Table — (DOCX) [file pone.0301759.s022.docx]

**S11 Table**. Constraint on the future annual temperature in North America for the period of 2015-2100 based on CMIP5 projections by using observed annual temperature growth rates.

|  | Observed annual temperature growth rates ± one standard deviation  (℃ year^-1^) |  | Future annual temperature growth rates  before emergent constraint | | Future annual temperature growth rates  after emergent constraint | | Underestimated future  temperature increase  (%) | Reduced uncertainty (%) |
| --- | --- | --- | --- | --- | --- | --- | --- | --- |
|  |  |  | Mean value  (℃ year^-1^) | one standard deviation | Mean value  (℃ year^-1^) | one standard deviation |  |  |
| HadCRUT4 | 0.0385 ± 0.0070 | RCP45 | 0.0307 | 0.0116 | 0.0327 | 0.0090 | 6.5% | 22.4% |
|  |  | RCP85 | 0.0680 | 0.0154 | 0.0726 | 0.0134 | 6.8% | 13.0% |
| NOAA | 0.0371 ± 0.0065 | RCP45 | 0.0307 | 0.0116 | 0.0317 | 0.0088 | 3.3% | 24.1% |
|  |  | RCP85 | 0.0680 | 0.0154 | 0.0711 | 0.0129 | 4.6% | 16.2% |
| GISS | 0.0422 ± 0.0070 | RCP45 | 0.0307 | 0.0116 | 0.0355 | 0.0094 | 15.6% | 19.0% |
|  |  | RCP85 | 0.0680 | 0.0154 | 0.0766 | 0.0134 | 12.6% | 13.0% |
| GHCN | 0.0403 ± 0.0070 | RCP45 | 0.0307 | 0.0116 | 0.0340 | 0.0091 | 10.7% | 21.6% |
|  |  | RCP85 | 0.0680 | 0.0154 | 0.0745 | 0.0133 | 9.6% | 13.6% |
