# Supplementary material for: Uncertainty reduction for precipitation prediction in North America
Source: PLoS One. 2024 May 22;19(5):e0301759. doi: 10.1371/journal.pone.0301759 (PMC11111050; doi:10.1371/journal.pone.0301759)
Supplement: S12 Table — (DOCX) [file pone.0301759.s023.docx]

**S12 Table**. Constraint on the future annual total evaporation (ET) growth rates in North America for the period of 2015-2100 based on CMIP6 projections by using the constrained future annual precipitation growth rates.

|  | Constrained future annual precipitation growth rates± one standard deviation  (mm year^-1^) | | Future annual ET growth rates  before emergent constraint | | Future annual ET growth rates  after emergent constraint | | Overestimated future ET increase  (%) | Reduced uncertainty (%) |
| --- | --- | --- | --- | --- | --- | --- | --- | --- |
|  |  |  | Mean value  (mm year^-1^) | one standard deviation | Mean value  (mm year^-1^) | one standard deviation |  |  |
| HadCRUT4 | SSP126 | 0.3028 ± 0.1227 | 0.1742 | 0.0849 | 0.1660 | 0.0640 | 4.7% | 24.6% |
|  | SSP245 | 0.6178 ± 0.1678 | 0.2604 | 0.2041 | 0.2226 | 0.1744 | 14.5% | 14.6% |
|  | SSP370 | 0.9834 ± 0.2834 | 0.4011 | 0.2929 | 0.3552 | 0.2586 | 11.4% | 11.7% |
|  | SSP585 | 1.2970 ± 0.3370 | 0.5858 | 0.3440 | 0.5204 | 0.3043 | 11.2% | 11.5% |
| NOAA | SSP126 | 0.2903 ± 0.1303 | 0.1742 | 0.0849 | 0.1613 | 0.0684 | 7.4% | 19.4% |
|  | SSP245 | 0.6012 ± 0.1712 | 0.2604 | 0.2041 | 0.2121 | 0.1699 | 18.5% | 16.8% |
|  | SSP370 | 0.9569 ± 0.2869 | 0.4011 | 0.2929 | 0.3375 | 0.2623 | 15.9% | 10.4% |
|  | SSP585 | 1.2691 ± 0.3591 | 0.5858 | 0.3440 | 0.5033 | 0.2991 | 14.1% | 13.1% |
| GISS | SSP126 | 0.3455 ± 0.1155 | 0.1742 | 0.0849 | 0.1819 | 0.0648 | -4.4% | 23.7% |
|  | SSP245 | 0.6748 ± 0.1548 | 0.2604 | 0.2041 | 0.2586 | 0.1659 | 0.7% | 18.7% |
|  | SSP370 | 1.0742 ± 0.2742 | 0.4011 | 0.2929 | 0.4160 | 0.2530 | -3.7% | 13.6% |
|  | SSP585 | 1.3926 ± 0.3326 | 0.5858 | 0.3440 | 0.5789 | 0.2890 | 1.2% | 16.0% |
| GHCN | SSP126 | 0.3126 ± 0.1226 | 0.1742 | 0.0849 | 0.1697 | 0.0656 | 2.6% | 22.7% |
|  | SSP245 | 0.6310 ± 0.1610 | 0.2604 | 0.2041 | 0.2309 | 0.1704 | 11.3% | 16.5% |
|  | SSP370 | 1.0044 ± 0.2644 | 0.4011 | 0.2929 | 0.3693 | 0.2519 | 7.9% | 14.0% |
|  | SSP585 | 1.3190 ± 0.3390 | 0.5858 | 0.3440 | 0.5339 | 0.2899 | 8.9% | 15.7% |
