# Supplementary material for: Uncertainty reduction for precipitation prediction in North America
Source: PLoS One. 2024 May 22;19(5):e0301759. doi: 10.1371/journal.pone.0301759 (PMC11111050; doi:10.1371/journal.pone.0301759)
Supplement: S13 Table — (DOCX) [file pone.0301759.s024.docx]

**S13 Table**. Constraint on the future annual total evaporation (ET) in North America for the period of 2015-2100 based on CMIP5 projections by using constrained future annual precipitation growth rates.

|  | Constrained future annual precipitation growth rates± one standard deviation  (mm year^-1^) | | Future annual ET growth rates  before emergent constraint | | Future annual ET growth rates  after emergent constraint | | Underestimated future ET increase  (%) | Reduced uncertainty (%) |
| --- | --- | --- | --- | --- | --- | --- | --- | --- |
|  |  |  | Mean value  (mm year^-1^) | one standard deviation | Mean value  (mm year^-1^) | one standard deviation |  |  |
| HadCRUT4 | RCP45 | 0.5587 ± 0.1386 | 0.2605 | 0.1024 | 0.2698 | 0.0698 | 3.6% | 31.8% |
|  | RCP85 | 1.0902 ± 0.2102 | 0.4009 | 0.1665 | 0.4215 | 0.1452 | 5.1% | 12.8% |
| NOAA | RCP45 | 0.5445 ± 0.1444 | 0.2605 | 0.1024 | 0.2631 | 0.0737 | 1.0% | 28.0% |
|  | RCP85 | 1.0661 ± 0.1961 | 0.4009 | 0.1665 | 0.4076 | 0.1373 | 1.7% | 17.5% |
| GISS | RCP45 | 0.5959 ± 0.1458 | 0.2605 | 0.1024 | 0.2875 | 0.0709 | 10.4% | 30.8% |
|  | RCP85 | 1.1533 ± 0.2132 | 0.4009 | 0.1665 | 0.4577 | 0.1494 | 14.2% | 10.3% |
| GHCN | RCP45 | 0.5761 ± 0.1460 | 0.2605 | 0.1024 | 0.2781 | 0.0714 | 6.8% | 30.3% |
|  | RCP85 | 1.1197 ± 0.2097 | 0.4009 | 0.1665 | 0.4384 | 0.1427 | 9.4% | 14.3% |
