# Supplementary material for: Uncertainty reduction for precipitation prediction in North America
Source: PLoS One. 2024 May 22;19(5):e0301759. doi: 10.1371/journal.pone.0301759 (PMC11111050; doi:10.1371/journal.pone.0301759)
Supplement: S14 Table — Note: The unconstrained water availability = the unconstrained precipitation–the unconstrained total evaporation; the constrained water availability = the constrained precipitation–the constrained total evaporation; Overestimated percentages = │constrained water availability–unconstrained water availability│/unconstrained water availability. (DOCX) [file pone.0301759.s025.docx]

**S14 Table**. **Water availability based on the constrained precipitation and the constrained total evaporation.** Note: the unconstrained water availability=the unconstrained precipitation－the unconstrained total evaporation; the constrained water availability=the constrained precipitation－the constrained total evaporation; Overestimated percentages=│constrained water availability－unconstrained water availability│/unconstrained water availability;

|  |  | Future annual water availability growth rates  before emergent constraint  (mm year^-1^) | Future annual water availability growth rates  after emergent constraint  (mm year^-1^) | Overestimated water availability  (mm year^-1^) | Overestimated percentages |
| --- | --- | --- | --- | --- | --- |
| HadCRUT4 | SSP126 | 0.1796 | 0.1368 | 0.0428 | 23.8% |
|  | SSP245 | 0.4439 | 0.3952 | 0.0487 | 11.0% |
|  | SSP370 | 0.6741 | 0.6282 | 0.0459 | 6.8% |
|  | SSP585 | 0.8506 | 0.7766 | 0.074 | 8.7% |
| NOAA | SSP126 | 0.1796 | 0.129 | 0.0506 | 28.2% |
|  | SSP245 | 0.4439 | 0.3891 | 0.0548 | 12.3% |
|  | SSP370 | 0.6741 | 0.6194 | 0.0547 | 8.1% |
|  | SSP585 | 0.8506 | 0.7658 | 0.0848 | 10.0% |
| GISS | SSP126 | 0.1796 | 0.1636 | 0.016 | 8.9% |
|  | SSP245 | 0.4439 | 0.4162 | 0.0277 | 6.2% |
|  | SSP370 | 0.6741 | 0.6582 | 0.0159 | 2.4% |
|  | SSP585 | 0.8506 | 0.8137 | 0.0369 | 4.3% |
| GHCN | SSP126 | 0.1796 | 0.1429 | 0.0367 | 20.4% |
|  | SSP245 | 0.4439 | 0.4001 | 0.0438 | 9.9% |
|  | SSP370 | 0.6741 | 0.6351 | 0.039 | 5.8% |
|  | SSP585 | 0.8506 | 0.7851 | 0.0655 | 7.7% |
