# Supplementary material for: Uncertainty reduction for precipitation prediction in North America
Source: PLoS One. 2024 May 22;19(5):e0301759. doi: 10.1371/journal.pone.0301759 (PMC11111050; doi:10.1371/journal.pone.0301759)
Supplement: S15 Table — (DOCX) [file pone.0301759.s026.docx]

**S15 Table. Full name of the CMIP6 models for collecting the daily data of precipitation during 2015-2100.**

|  | SSP126 | SSP245 | SSP370 | SSP585 |
| --- | --- | --- | --- | --- |
| 1 | ACCESS-ESM1-5 | BCC-CSM2-MR | ACCESS-CM2 | ACCESS-CM2 |
| 2 | CESM2 | CESM2 | CESM2 | CESM2 |
| 3 | CNRM-ESM2-1 | CESM2-WACCM | CNRM-ESM2-1 | CESM2-WACCM |
| 4 | HadGEM3-GC31-LL | INM-CM4-8 | INM-CM4-8 | HadGEM3-GC31-LL |
| 5 | INM-CM4-8 | INM-CM5-0 | INM-CM5-0 | INM-CM4-8 |
| 6 | INM-CM5-0 | IPSL-CM6A-LR | IPSL-CM6A-LR | INM-CM5-0 |
| 7 | IPSL-CM6A-LR | MPI-ESM1-2-LR | MPI-ESM1-2-LR | IPSL-CM6A-LR |
| 8 | MIROC6 | NorESM2-LM | NorESM2-MM | NorESM2-LM |
| 9 | MPI-ESM1-2-LR | NorESM2-MM | UKESM1-0-LL | NorESM2-MM |
| 10 | NorESM2-LM | UKESM1-0-LL |  | UKESM1-0-LL |
| 11 | NorESM2-MM |  |  |  |
| 12 | UKESM1-0-LL |  |  |  |
